# Genome-wide dissection of heterosis for yield traits in two-line hybrid rice populations

**DOI:** 10.1038/s41598-017-06742-7

**Published:** 2017-08-09

**Authors:** Gang Zhen, Peng Qin, Kai Yu Liu, Dong Yang Nie, Yuan Zhu Yang, Xing Wang Deng, Hang He

**Affiliations:** 1 State Key Laboratory of Protein and Plant Gene Research, Peking-Tsinghua Center for Life Sciences, School of Advanced Agriculture Sciences and School of Life Sciences, Peking University, Beijing, China; 2 Ava Seed Academy of Sciences, Changsha, Hunan China

## Abstract

Heterosis has been widely utilized in agriculture and is important for world food safety. Many genetic models have been proposed as mechanisms underlying heterosis during the past century, yet more evidence is needed to support such models. To investigate heterosis in two-line hybrid rice, we generated a partial diallel crossing scheme, which consisted of approximately 500 F1 hybrids derived from 14 male sterile lines and 39 restorer lines. In this population, increased panicle number played the most important role in yield heterosis of hybrid rice. Genome-wide association studies identified many QTLs related to the yield traits of F1 hybrids, better paternal heterosis and special combining ability. Relevant genes, including *Hd3a*, *qGL3*, *OsmiR156h*, and *LAX2*, were identified as candidates within these QTLs. Nearly forty percent of the QTLs had only two genotypes in the F1 hybrids, mainly because the maternal lines were under intense selective pressure. Further analysis found male sterile lines and restorer lines made different superior allele contributions to F1 hybrids, and their contributions varied among different traits. These results extend our understanding of the molecular basis of heterosis in two-line hybrid rice.

## Introduction

Asia produces more than 90% of rice, which supplies one quarter of the total calories consumed by the world population and is the main food for most of the world’s poor^1^. In China, rice production occupies 18.3% of total farmland (corn occupies 24.4%); hybrid rice was sown in approximately 12.8 million ha in 2014, whereas conventional rice was sown in approximately 11.1 million ha (Crop Seed Industry Development Report in China (2015)). In 1973, a three-line hybrid rice system composed of one cytoplasmic male sterile line, one maintainer line, and one restorer line was successfully developed in China^2^. Then in the 1980s, a two-line hybrid system, composed of one environment-sensitive genic male sterile (EGMS) line and one restorer line, was developed^3^. The two-line system has several advantages over the three-line system, including easy male sterile line multiplication (no maintainer line is needed for reproduction of male sterile lines), no restriction with regard to the restorer line (theoretically, all cultivars with normal pollen can be used as restorer lines), and easy use of inter-subspecific heterosis (easy introduction of a wide range of genes into the EGMS line rather than into the cytoplasmic male sterile line)^4^. While commercial hybrid rice is estimated to outperform conventional inbred rice by >20% in grain yield, two-line commercial hybrid rice is estimated to outperform its three-line counterpart by ~10% in grain yield^4^. Therefore, the two-line hybrid rice system has become increasingly important in hybrid rice breeding^5^.

Although heterosis, or hybrid vigor, has been successfully used in hybrid rice production, as well as in production of many other crop species, including corn and sorghum, its genetic mechanism remains unclear^6–8^. Since George H. Shull rediscovered heterosis in 1908^9^, many hypothetical genetic mechanisms, including dominance^9–11^, overdominance^12, 13^, epistasis^14–16^, gene balance^17, 18^, and protein quality control^19, 20^ have been proposed to explain heterosis. Today, most heterosis studies mainly focus on important agronomic traits in crops such as corn and rice. Due to the quantitative nature of these traits, many genetic mechanisms likely function in heterosis; therefore, it is probable that no single genetic mechanism can adequately explain all aspects of the heterosis phenomenon^7, 21^.

In the past twenty years, genetic mapping of the loci underlying rice heterosis using molecular markers has been performed^22–28^. The accuracy of early genetic mapping suffered from the use of low-density markers; for example, the mapping resolution was low and did not allow differentiation between overdominance and pseudo-overdominance. Most of these studies used parental materials derived from bi-parental mapping populations, and the genetic diversity among these parental materials was very low. Therefore, the genetic mechanisms derived from studies of these populations might be of little relevance to actual hybrid rice production, as many genetically diversified male sterile lines and restorer lines are currently used in hybrid rice breeding. With the rapid development of genome sequencing, genome-wide association study (GWAS) using high-density genetic markers has been widely used to dissect the genetic mechanisms underlying quantitative traits in crop species^29^. In rice, GWAS has proven to be a useful tool for identifying important genes related to agronomic traits^30, 31^. Recently, Huang *et al*. genotyped 10,074 F2 lines derived from 17 representative varieties from 3 different hybrid rice systems, revealing many important genes related to 7 yield-related traits^32, 33^. These studies indicate that genetic mapping in a multi-parental population using high-density markers could be utilized to discover the genetic basis of heterosis.

Although middle-parent heterosis (superior performance of the F1 hybrid in comparison with the average of both parents) was of great interest in previous heterosis analysis, better-parent heterosis (superior performance of the F1 hybrid in comparison with the better parent) is the major goal underlying the wide adoption of hybrid techniques in agriculture because of its economic impact^34^. In hybrid rice, better paternal heterosis (superior performance of the F1 hybrid in comparison with the male parent) is most important to breeders because the female parent is sterile. Special combining ability (SCA), a very important indicator when selecting for superior hybrid cultivars in rice breeding, is mainly affected by non-additive effects such as dominance and epistasis^35^. Identification of the genetic mechanisms underlying better paternal heterosis and SCA is of practical importance in hybrid rice production.

In hybrid rice breeding, male sterile lines and restorer lines are under quite different selective pressures. In addition to sterility, male sterile lines have to be dwarfed (facilitating pollination), show early-heading (short growing period), and have good combining abilities for many agronomic traits. Therefore, the breeding of male sterile lines requires much more effort than that of restorer lines. In addition, male sterile lines have a demographic history quite different from that of restorer lines, as the former are all derived from several main ancestors, while the latter have a much broader genetic origin. Therefore, the genetic architectures underlying many traits in male sterile lines are probably quite different from those that underlie the same traits in restorer lines, and these differences may have distinct impacts on the agronomic performance of F1 hybrids.

In this study, we constructed a partial diallel two-line hybrid rice cross scheme and measured heterosis in nine yield-related traits. We performed GWAS and identified genetic loci underlying better paternal heterosis and SCA in the hybrid rice lines. We found that the superior allele ratios of many QTLs differed markedly between the male sterile lines and restorer lines. Furthermore, we screened for genetic regions under selective pressure in both male sterile lines and restorer lines, revealing the role of selective pressure in the heterosis phenomenon in F1 hybrids. All the genotype and phenotype data used in this study is provided in Supplementary Data S1 and S2.

## Results

### Heterosis varied among different traits in two-line hybrid rice lines, and increased panicle number contributed most to yield

**Figure 1:**
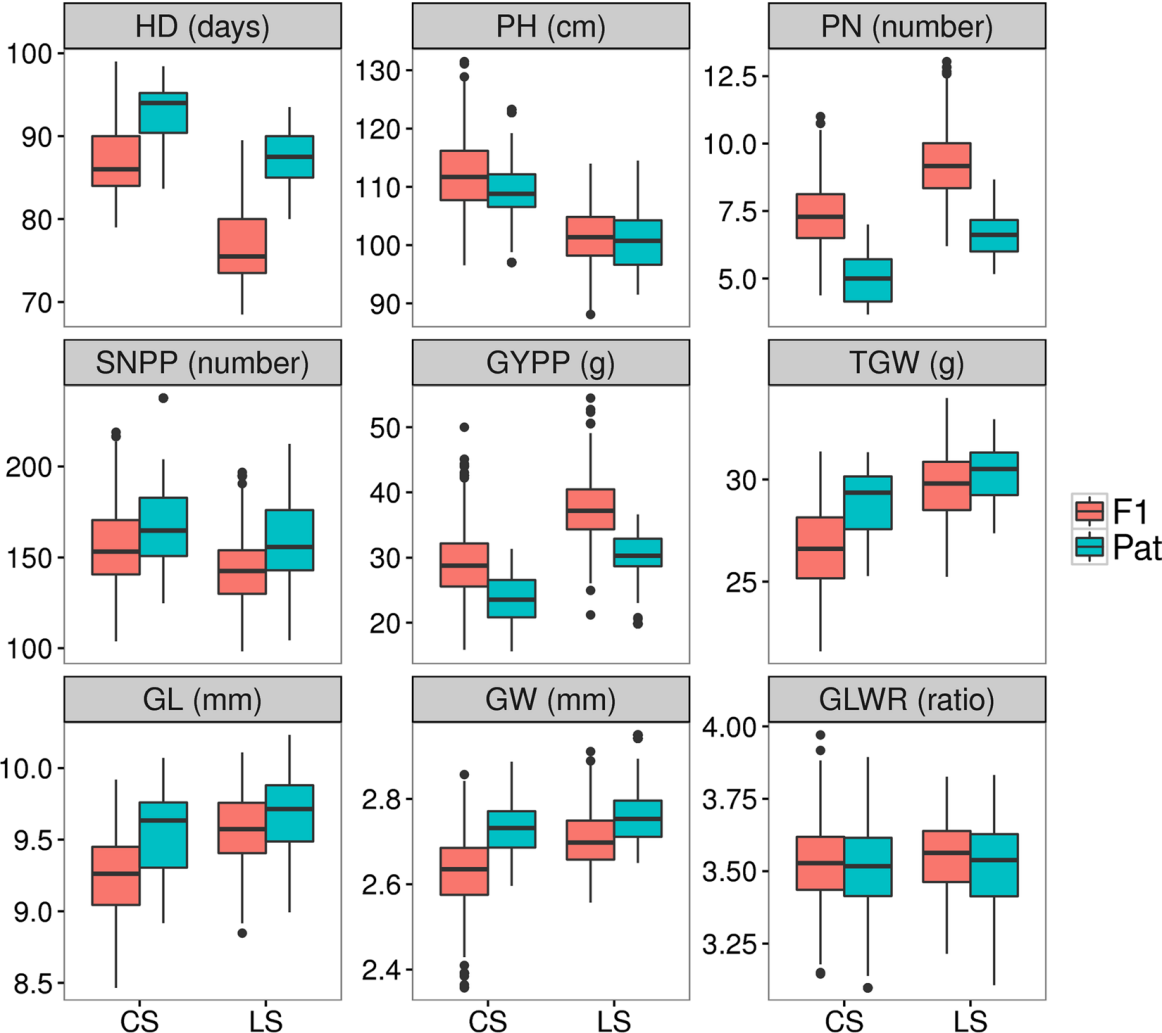
Phenotype distributions of nine agronomic traits in F1 hybrids (F1) and their male parents (Pat).

In this experiment, fourteen photo-thermo-sensitive genic male sterile (PTGMS, one type of EGMS) lines that have been widely used in commercial hybrid rice breeding in China were used as maternal lines, and three core recombinant inbred lines (RILs) (each consisting of 12–14 lines) were used as restorer (paternal) lines. Each restorer line was crossed to all 14 male sterile lines, yielding a partial diallel cross panel consisting of 500 F1 hybrids. Field experiments were first performed in Changsha (CS), China, in the summer of 2014, after which they were performed in Lingshui (LS), China, in the spring of 2015. Nine agronomic traits (heading date (HD), plant height (PH), panicle number per plant (PN), seed number per panicle (SNPP), grain yield per plant (GYPP), 1000 grain weight (TGW), grain length (GL), grain width (GW), and grain length/width ratio (GLWR)) were evaluated. All nine traits showed a continuous distribution (Supplementary Figs. S1–S9), indicating the presence of complex underlying genetic mechanisms. In commercial hybrid rice breeding, it is impossible to compare the grain yield-related traits of F1 hybrids to their corresponding middle parent values (MPVs) because the maternal lines are sterile; thus, in this study, we used better paternal value (BPaV) to measure heterosis in F1 hybrids. We found that heterosis in the two-line hybrid system varied among traits (Fig. 1 and Supplementary Fig. S10). Most F1 hybrids had an earlier HD (91.1% in the CS dataset, and 99.0% in the LS dataset) and increased PH (58.5% for CS, and 54.4% for LS) in comparison with those of their paternal parents. For PN and GYPP, most F1 hybrids (90.9% for CS and 94.6% for LS for PN, 78.6% in CS and 88.1% in LS for GYPP) showed performance better than that of their paternal parents. However, for SNPP and grain shape-related traits (TGW, GL, and GW), most F1 hybrids (average: 77.8% for CS and 63.4% for LS) had performance worse than that of their male parents. We evaluated some traits of the male sterile lines in the LS dataset, and strong hybrid vigor was also observed when maternal effects were taken into account. For example, when compared to MPVs, most F1 hybrids showed an earlier HD (95.2%), as well as increased PH (100.0%) and TGW (87.5%) (Supplementary Fig. S11).

Grain yield is mainly determined by three components: PN, SNPP, and TGW. Previous phenotype comparisons have shown that most F1 hybrids have higher GYPP and PN in comparison with those of their male parents, as well as lower SNPP and TGW (Fig. 1 and Supplementary Fig. S10). Phenotype correlation analysis showed that the phenotypic correlation between PN and GYPP (r = 0.482 for CS and r = 0.64 for LS) was higher than that between SNPP and GYPP (r = 0.476 for CS and r = 0.35 for LS), as well as that between TGW and GYPP (r = 0.31 for CS and r = −0.02 for LS) (Supplementary Fig. S12). Linear regression analysis showed that the grain yield variance explained by PN (0.28 for CS and 0.42 for LS) was much larger than that explained by any of the other yield-related traits for CS and LS (Supplementary Fig. S13). These results indicate that increased panicle number is more important than the other tested traits with regard to boosting the grain yield production of F1 hybrids.

### Genotypic variation among parental lines and population structure in F1 hybrids

**Figure 2:**
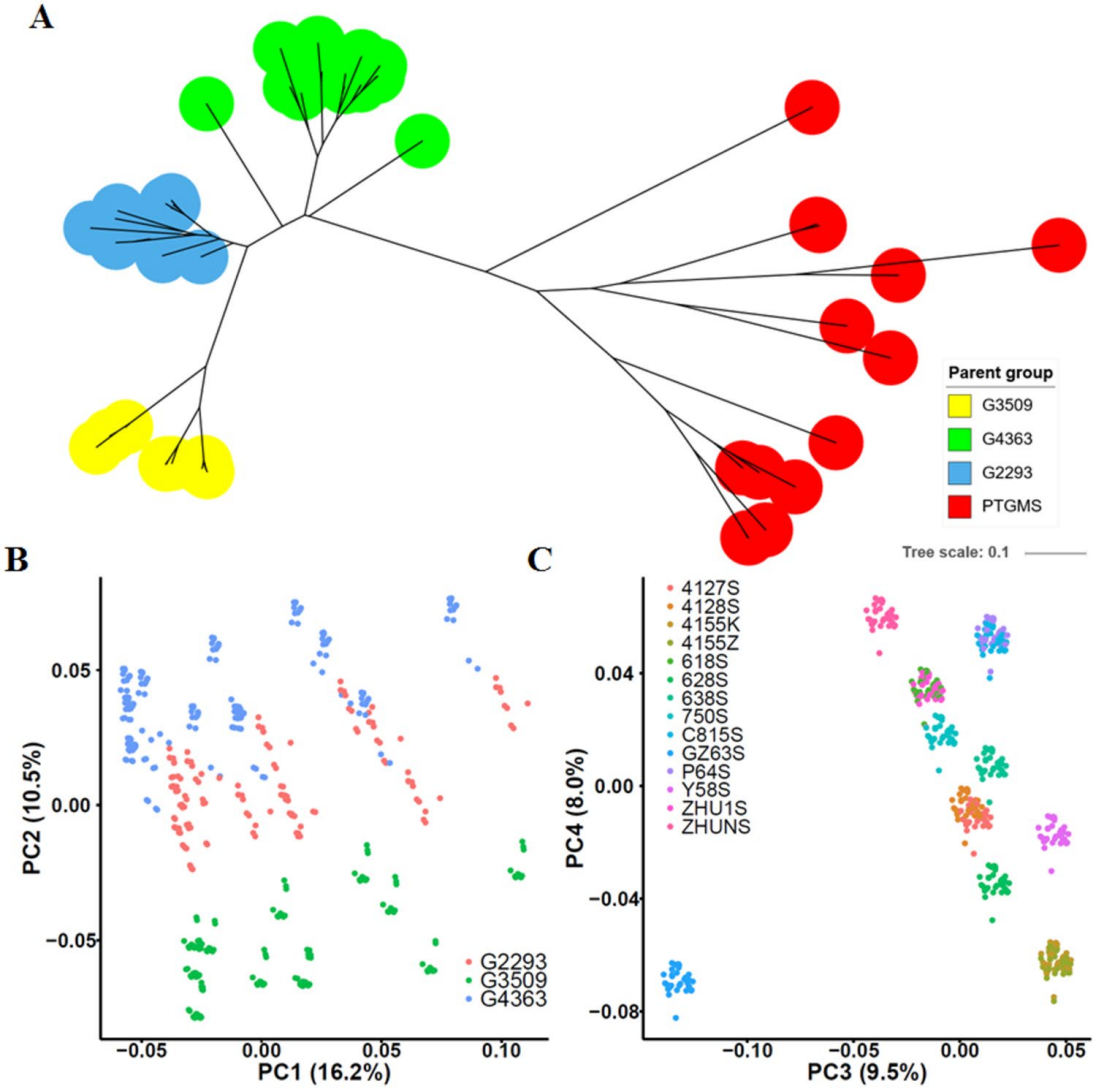
Phylogenetic relationships among parental lines (**A**) and population structure among F1 hybrids (**B,C**).

We genotyped all 53 parents using the rice 50 K SNP Chip on the Illumina Infinium platform^36^. After quality control, 26920 polymorphic SNPs remained for further analysis. To assess genetic differences among parental lines, phylogenetic analysis was performed using filtered SNP genotypes. All parents were separated into two highly divergent clusters: male sterile lines and restorer lines (Fig. 2A). The restorer lines were further separated into three groups in accordance with their pedigree information. It should be noted that the male sterile lines were also highly divergent as measured by branch length, and this reflected the complicated breeding history of each PTGMS.

The genotype for each F1 hybrid was obtained by combining the genotypes of its inbred parents. All F1 hybrids had high average genome heterozygosity, ranging from 17.5% to 28.4%, with a median of 23.1% (Supplementary Fig. S14). By calculating the average heterozygosity in 100-kb windows across the whole genome, high heterozygosity was observed along the whole genome except for the regions around centromeres (Supplementary Fig. S14). Principal component analysis (PCA) revealed highly-structured genetic relationships among the F1 hybrids (Fig. 2B and C). The first and second genomic PCs mainly separated the F1 hybrids into 3 groups consistent with their male parental origins, while the third and fourth PCs separated them into many small groups consistent with their female parental origins.

### Genome-wide association studies

To deepen our understanding of the genetic basis of heterosis in two-line hybrid rice populations, we conducted GWAS analysis in three categories: F1 GWAS, BPaV GWAS, and SCA GWAS. In brief, the original F1 hybrid phenotypes were used in the F1 GWAS, while BPaV and SCA were treated as phenotypes in the BPaV GWAS and SCA GWAS. Because the study panel was highly structured, familial relationships among F1 hybrids could impair interpretation of association analysis results, as long-range correlations among genetic markers might cause false positive signals, which can be located on another chromosome^37–39^. Thus, we adopted the forward-selection resampling GWAS approach first proposed by Valdar *et al*.^40^ and successfully used in association analysis of heterogeneous stock mice^38, 41, 42^ and maize nested association mapping populations^43–46^.

**Figure 3:**
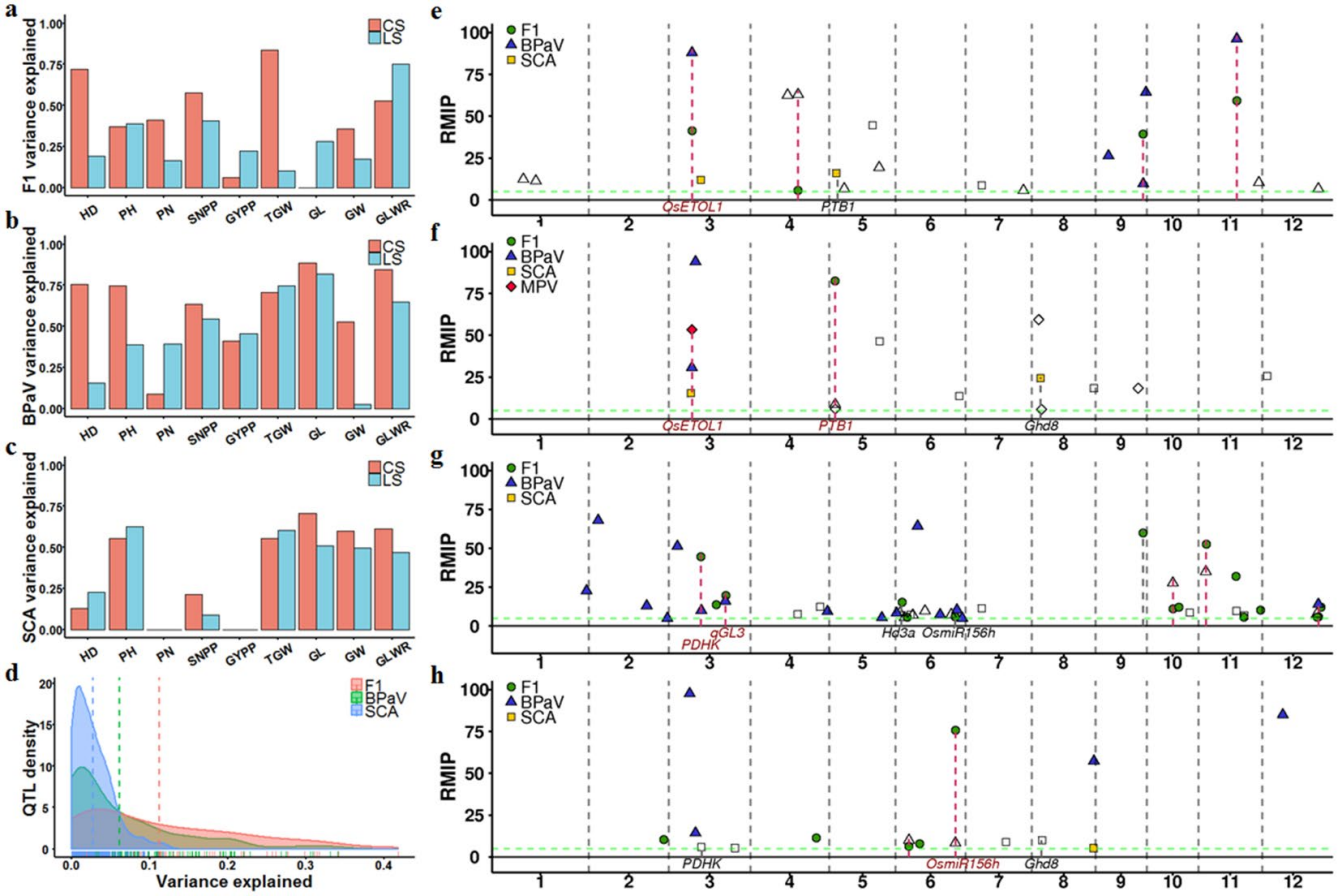
GWAS results. (**a**) Total original phenotype variance of F1 hybrids explained by QTLs identified in F1 GWAS. (**b**) Total BPaV variance explained by QTLs identified in BPaV GWAS. (**c**) Total SCA variance explained by QTLs identified in SCA GWAS. (**d**) Frequency distribution of QTLs identified in F1 GWAS, BPaV GWAS, and SCA GWAS based on the variance explained by each QTL. The vertical dashed lines represent the average variance explained for the three QTL groups. (**e,f**) Manhattan plots of HD GWAS results for the CS dataset (E) and LS dataset (F). (**g,h**) Manhattan plots of SNPP GWAS results for the CS dataset (**g**) and LS dataset (**h**). Y-axis, RMIP in resample GWAS; red dashed lines, shared QTLs among different GWAS categories; open symbols, GWAS signals disappeared when using corresponding original F1 hybrid phenotypes as covariates.

A brief summary of all GWAS results is shown in Table 1. F1 GWAS identified several quantitative trait loci (QTLs, numbers ranging from 0 to 16, with an average of 4.1) for most traits (all except SNPP and TGW) in the CS dataset, and most of these traits had <50% of their total phenotype variance explained (Fig. 3a). BPaV GWAS identified more association signals (ranging from 1 to 35, with an average of 11.2) than did F1 GWAS, and a high proportion (>60%) of BPaV variance was explained by these QTLs for most traits (Fig. 3b). For SCA GWAS, dozens of QTLs were identified for PH and grain shape-related traits (TGW, GL, GW, and GLWR, ranging from 18 to 37, with an average of 24.2), and >50% of the SCA variance for these traits was explained (Fig. 3c). Many important genes overlapped these trait-associated regions (Supplementary Data S3). Most QTLs appeared at a low frequency (measured as resample model inclusion probability, RMIP) in the GWAS analysis (Supplementary Fig. S15). After assessing QTL effects (measured in phenotype variance explained by their lead SNPs), we found that a large proportion of QTLs identified fin F1 GWAS (45.7% for CS and 40.7% for LS) were large-effect QTLs that explained >10% of the variance of their original phenotypes, while this percentage was much lower in the GWAS results for BPaV (23.5% for CS and 20.2% for LS) and SCA (1.7% for CS and 1.4% for LS) (Fig. 3d). These results suggest that both better paternal heterosis and SCA are controlled by many loci with small effects.

**Table 1:** Summary of QTLs found in GWAS.

Dataset	Category	HD	PH	PN	SNPP	YPP	TGW	GL	GW	GLWR
Changsha	F1 GWAS	4	1	6	16	1	14	0	3	1
	BPaV GWAS	14	6	3	23	8	6	23	3	16
	SCA GWAS	4	28	0	7	0	19	18	21	19
Linshui	F1 GWAS	1	1	1	5	7	1	2	2	7
	BPaV GWAS	3	2	9	6	24	35	15	1	4
	SCA GWAS	6	37	0	5	0	22	23	34	21
	MPV GWAS	5	2	NA	NA	NA	6	6	4	1

There were few overlaps between the QTLs identified via F1 GWAS and those found via BPaV and SCA GWAS (Fig. 3e–h and Supplementary Figs S16–S22). F1 GWAS revealed 46 association signals in the CS dataset and 27 association signals in the LS dataset. Among these regions, 13 of the association signals identified in the CS dataset and 11 of those identified in the LS dataset overlapped with the corresponding BPaV GWAS results, while only 3 of those identified in the CS dataset and 2 of those identified in the LS dataset overlapped with the SCA GWAS results. Among all traits, high overlap rates were only found between the F1 GWAS and BPaV GWAS results for HD and SNPP. Many important genes related to relevant biological processes were identified as candidates within these overlapping regions.

For HD, only one QTL (lead SNP: F0921300849AC) was identified by the F1 GWAS in the CS dataset when the additive inheritance model was used. This QTL explained 41.8% of the HD variance in the CS dataset. Another QTL (lead SNP: R0921365484AG), located approximately 65 kb downstream of F0921300849AC, was found in the BPaV GWAS of HD for the CS dataset, and it explained 30.8% of the HD_BPaV (representing the BPaV of HD in the F1 hybrids) variance. These two lead SNPs were in strong linkage disequilibrium (r^2^ = 0.94), suggesting the same association signal in the F1 GWAS and BPaV GWAS. F1 GWAS (CS) using other inheritance models detected three more QTLs located on chromosomes 3, 4, and 11. A QTL located on chromosome 3 and harboring *OsETOL1*, which encodes a protein that controls ethylene biosynthesis and spikelet fertility^47^, was also discovered via the BPaV, SCA, and MPV GWAS of HD for the LS dataset (Fig. 3f). These three QTLs were also found in the BPaV GWAS of HD for the CS dataset. The F1 GWAS of HD for the LS dataset only identified one QTL located on chromosome 5 (lead SNP: R0502670171GA, RMIP = 82.3), while this QTL was also discovered at a low frequency via the BPaV GWAS (RMIP = 8.7) and MPV GWAS (RMIP = 6.0) (Fig. 3f). *PTB1*, which encodes a RING-type E3 ubiquitin ligase that regulates the panicle seed setting rate of rice by promoting pollen tube growth^48^, was identified as a candidate gene within this region. The SCA and MPV GWAS of HD for the LS dataset identified a region located on chromosome 8 that overlapped with *Ghd8*, which promotes flowering under short-day conditions^49^.

The F1 GWAS of SNPP for the CS dataset identified 16 QTLs. The lead SNP, F0921300849AC was also detected in the F1 GWAS of HD for the CS dataset, and explained the largest proportion of SNPP variance (31.7%) (Fig. 3g). The F1 GWAS of SNPP for the CS dataset also identified one QTL located on chromosome 6. *Hd3a*, which encodes a protein that controls rice-flowering through interactions with OsFD1 and 14-3-3^50^, was identified as a candidate gene within this region. The BPaV GWAS of SNPP for the CS dataset identified 23 QTLs. The lead SNP, F1225114400AC, explained the largest fraction of SNPP_BPaV variance (16%). Five genomic regions (shorter than 250 kb) located on chromosomes 3, 10, 11, and 12 were shared by the F1 GWAS and BPaV GWAS results. However, all of these regions had differently located association signals, and only one of them (on chromosome 10) showed high linkage (r^2^ = 0.87) between the two lead SNPs identified by the F1 GWAS and BPaV GWAS. The F1 GWAS and BPaV GWAS of SNPP for the LS dataset each identified 5 and 6 QTLs, among which 2 QTLs (lead SNPs: R0605948297AG and R0626712051CA) were shared by both sets of results (Fig. 3h). Lead SNP R0605948297 explained 14.4% of the variance in SNPP and 10.8% of the variance in SNPP_BPaV. Lead SNP R0626712051CA explained 21.7% of the variance in SNPP and 11.7% of the variance in SNPP_BPaV. This region was also identified by the F1 GWAS of SNPP for the CS dataset. *OsmiR156h*, which affects rice tillering^51^, was identified as a candidate gene.

The GWAS results (QTL numbers, QTL effects, and overlapping rates) suggest that the genetic architectures underlying BPaV and SCA might be quite different from those underlying the original F1 hybrid phenotypes, and the QTLs identified in the BPaV and SCA GWAS might not directly affect the original F1 hybrid phenotypes. We re-ran the BPaV and SCA GWAS with the original F1 hybrid phenotypes added as covariates. This pleiotropy analysis aimed to remove the impact of QTLs directly influencing the original F1 hybrid phenotypes and to identify QTLs independently affecting BPaV or SCA^52^. For HD, the previous BPaV GWAS for the CS dataset identified 14 QTLs, of which 4 were shared by the F1 GWAS results. After using HD_F1 as a covariate, only 5 of the 14 QTLs remained in the new BPaV GWAS results, including 3 of the 4 overlapping QTLs, but not the QTL located on chromosome 4 (Fig. 3e). This finding confirms that these three QTLs directly influence the HD performance of the F1 hybrids and contribute to the better paternal heterosis of HD. However, among the 7 co-localized regions (5 in the CS dataset and 2 in the LS dataset) shared by the F1 GWAS and BPaV GWAS of SNPP, 4 regions were not present in the new BPaV GWAS results, including the region with high linkage in the CS dataset and two overlapping QTLs in the LS dataset (Fig. 3g and h). This finding indicates that these 3 shared regions affect SNPP heterosis by directly influencing the SNPP phenotypes of the F1 hybrids in a case of mediated pleiotropy^52^. When the original F1 hybrid phenotypes were used as covariates, 60.1% of the CS QTLs and 60.3% of the LS QTLs identified in the previous BPaV and SCA GWAS were present in the new sets of results. Larger proportions of QTLs were removed from the SCA GWAS results (46.6% for CS and 43.9% for LS) in comparison with those removed from the BPaV GWAS results (32.4% for CS and 33.3% for LS). The QTLs that were removed from the new GWAS analysis occurred at low frequencies in the previous BPaV and SCA GWAS (Supplementary Fig. S23). These results suggest that BPaV and SCA shared different genetic architectures with the original F1 hybrid phenotypes.

### Non-additive effects played more important roles than additive effects in three-genotype QTLs

**Figure 4:**
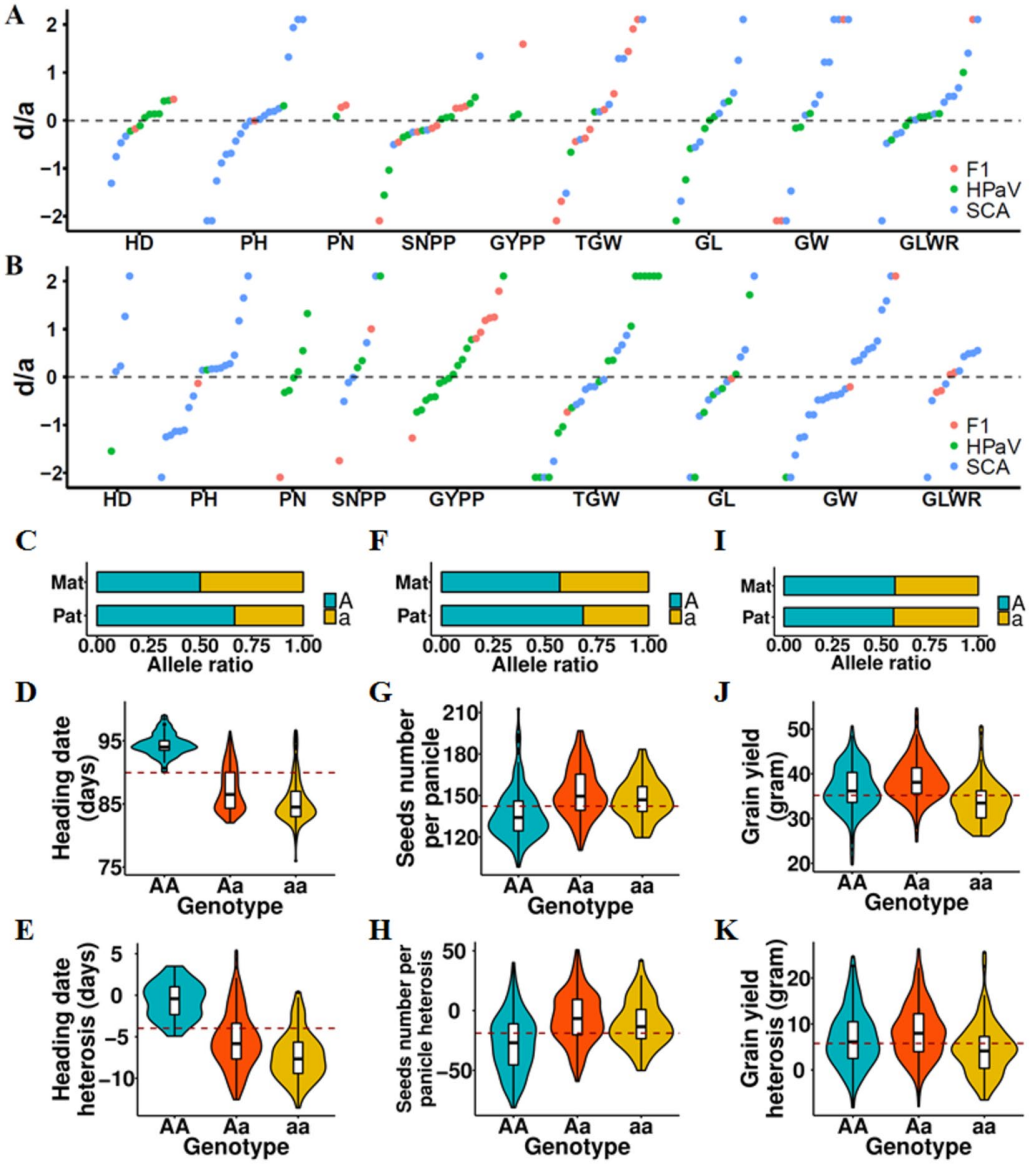
Non-additive effects played important roles in rice heterosis. (**A,B**) Dominance/additive (d/a) effects for all three-genotype QTLs identified in the CS (**A**) and LS (**B**) datasets. (**C**) Allele ratios of the QTL (lead SNP: F0921300849AC) in both parental groups (A: major allele; a: minor allele). (**D,E**) HD (**D**) and HD_BPaV (**E**) distributions for the QTL F0921300849AC. (**F**) Allele ratios of the QTL harboring *OsmiR156h* in both parental groups. (**G,H**) SNPP (**G**) and SNPP_BPaV (h) distributions for the QTL harboring *OsmiR156h*. (i) Allele ratios of the QTL harboring *MSP1* in both parental groups. (**J,K**) GYPP (j) and GYPP_BPaV (k) distributions for the QTL harboring *MSP1*. The red dashed lines in d, e, g, and h represent the mean values for the averages of the two homozygotes.

Many genetic models, including dominance and overdominance, have been proposed to explain the genetic basis of heterosis. To assess such effects for each QTL requires phenotype comparisons among all three genotypes (AA, Aa, and aa) in F1 hybrids. However, not all QTLs had all three genotypes in the F1 hybrids assessed in this study. For the F1 GWAS results, most of the QTLs (76.1% for CS and 85.2% for LS) had all three genotypes in the F1 hybrids, while the others had only two genotypes (Aa and either AA or aa). For the BPaV GWAS results, approximately half of the QTLs (44.1% for CS and 55.6% for LS) had all three genotypes. For the QTLs underlying SCA, 61.2% of CS QTLs and 52.7% of LS QTLs had all three genotypes. Therefore, we separated all QTLs into two groups, a three-genotype group and a two-genotype group, according to their genotype varieties in the F1 hybrids. For QTLs in the three-genotype group, a linear regression model was used to assess the relative contributions of dominance and additive effects for each QTL, revealing that most three-genotype QTLs (70.1% for CS and 82.5% for LS) showed non-additive effects, indicating the predominant roles of non-additive effects in heterosis (Fig. 4A and B). Many QTLs, especially those related to grain shape-related traits, showed over-dominance effects (28.6% for CS and 29.2% for LS, Supplementary Fig. 24). Many QTLs harboring important genes related to relevant biological processes showed non-additive effects. For example, the large effect QTL located on chromosome 9 (Fig. 3e) showed negative partial-dominance effects for both HD and HD_BPaV (Fig. 4C–E), which is in concordance with the reduced heading date of F1 hybrids compared to their mid-parent values (Supplementary Fig. S11). Another large effect QTL, located on chromosome 6 and harboring *OsmiR156h* (Fig. 3g and h), showed a positive dominance effect for SNPP, while it showed a positive over-dominance effect for SNPP_BPaV (Fig. 4F–H). The other large effect QTL, located on chromosome 1 and harboring *MSP1* (Supplementary Fig. S19), showed a positive dominance effect for GYPP, while it showed a positive partial-dominance effect for GYPP_BPaV (Fig. 4I–K).

Two-genotype QTLs were further separated into two groups according to the relative performance of heterozygotes and homozygotes. When the average phenotype value of heterozygotes (Aa) outperformed that of homozygotes (AA or aa), the two-genotype QTL was placed into the hetero-superior group; otherwise, the QTL was placed into the homo-superior group. The hetero-superior and homo-superior groups had nearly equal numbers of two-genotype QTLs for the BPaV GWAS results (27/30 for CS and 22/22 for LS). However, for the SCA GWAS results, the hetero-superior group outnumbered the homo-superior group by 32/13 in the CS dataset and 40/30 in the LS dataset.

### Male sterile lines and restorer lines made different superior allele contributions to F1 hybrids that varied among different traits

**Figure 5:**
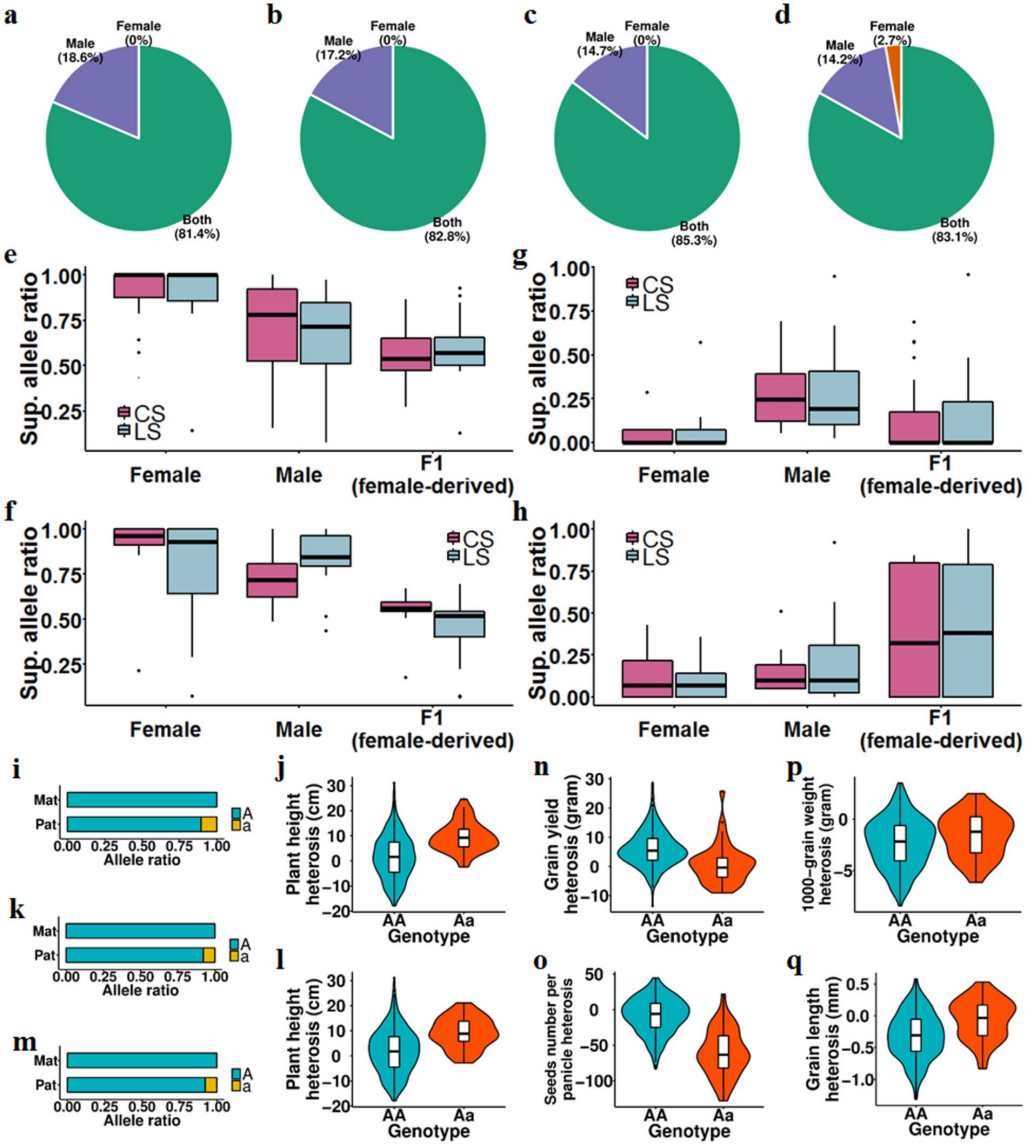
Male sterile lines and restorer lines made different superior allele contributions to F1 hybrids. (**a–d**) Superior allele contributions from either parental group for QTLs identified in the BPaV GWAS (a for CS, b for LS) and SCA GWAS (c for CS, d for LS). Two-genotype QTLs were separated into three groups: those with superior alleles contributed only by the restorer lines (male), only by the male sterile lines (female), and by both parental groups (both). (**e–h**) Superior allele ratios of male sterile lines, restorer lines, and F1 hybrids in homo-superior QTLs (e for BPaV GWAS results, f for SCA GWAS results) and hetero-superior QTLs (g for BPaV GWAS results, h for SCA GWAS results). (**i**) Allele ratios of the QTL harboring *OsGLU1* in both parental groups. (**j**) PH_BPaV distributions of the QTL harboring *OsGLU1*. (**k**) Allele ratios of the QTL harboring *OsPDK1* in both parental groups. (**l**) PH_BPaV distributions of the QTL harboring *OsPDK1*. (**m**) Allele ratios of the QTL harboring *qGL3* in both parental groups. (n-q) GYPP_BPaV (**n**), SNPP_BPaV (**o**), TGW_BPaV (**p**), and GL_BPaV (**q**) distributions of the QTL harboring *qGL3*.

In our crossing design, we can easily trace the parental origin of superior alleles for each QTL and measure the superior allele contributions from both parents. For large proportions of QTLs (ranging from 14.2% to 18.6%, Fig. 5a–d), superior alleles were contributed solely by restorer lines, indicating that restorer lines play an important role in heterosis by introducing new superior alleles. Next, the superior allele ratios in both parental groups and F1 hybrids were measured. Interestingly, we found that the two-genotype QTLs in the homo-superior group had much higher superior allele ratios in both male sterile lines and restorer lines (87.6% and 73.2% for CS, respectively; 84.2% and 72.3% for LS, respectively; Fig. 5e and f) in comparison with those of the two-genotype QTLs in the hetero-superior group (8.8% and 22.8% for CS, respectively; 8.0% and 22.7% for LS, respectively; Fig. 5g and h). The difference in the male sterile lines was much clearer than that in the restorer lines; more than half of the QTLs reached fixation in the male sterile lines. In the F1 hybrids, the male sterile lines provided large proportions of superior alleles for homo-superior QTLs (54.2% for CS and 53.1% for LS) (Fig. 5e and f), while the restorer lines provided most superior alleles for hetero-superior QTLs (72.7% for CS and 69.7% for LS) (Fig. 5g and h).

To validate our results, we genotyped another 36 male sterile lines and 48 restorer lines (Supplementary Fig. S25) that have been widely used in commercial hybrid rice breeding in China, after which superior allele ratios between the two QTL groups were measured. Contrasting superior allele ratios between the hetero-superior and homo-superior groups were also found in the new dataset (Supplementary Fig. S26). Among 81 (CS, representing 104 QTLs) and 85 (LS, representing 108 QTLs) two-genotype associated signals, 53 and 60 has low MAF (<0.05) in either male sterile lines or restorer lines in the previous parental dataset, while 59 and 58 has low MAF in the new parental dataset. Besides, among the 53 (CS) and 60 (LS) associated signals with low MAF in the previous parental dataset, 41 and 47 also has low MAF in the same parental lines in the new dataset. These results indicate that this phenomenon is common in hybrid rice parental lines.

The roles played by hetero-superior and homo-superior two-genotype QTLs in heterosis differed among traits. For example, the BPaV GWAS of PH for the CS dataset found 6 QTLs, among which 5 QTLs had only two genotypes in the F1 hybrids. Among these 5 two-genotype QTLs, four were hetero-superior, while the restorer lines contributed most of their superior alleles (average: 78.8%). This result is in concordance with the fact that the male sterile lines are all dwarfs due to strong artificial selection. Genes controlling rice height, including *OsGLU1* (Fig. 5i and j) and *OsPDK1* (Fig. 5k and l), were identified as candidates within these QTLs. The BPaV GWAS of SNPP and GYPP for the CS dataset identified 20 and 8 QTLs, respectively, among which 13 QTLs and 6 QTLs, respectively, were two-genotype QTLs. Among the two-genotype QTLs for SNPP and GYPP, many large-effect QTLs (4 related to SNPP_BPaV, each of which explained >7% variance, and 2 related to GYPP_BPaV, each of which explained >3.5% variance) were homo-superior. The superior alleles in these QTLs had reached fixation in the male sterile lines, and 64.4% of the superior alleles in the F1 hybrids were contributed by the male sterile lines. *qGL3*, which encodes OsPPKL1, a protein that controls grain length, grain weight, and grain yield^53^, was identified as a candidate gene within one of these QTLs (lead SNP: F0325216735GA; located on chromosome 3). In addition to SNPP_BPaV and GYPP_BPaV, we also found this QTL to be associated with TGW_BPaV (Supplementary Fig. S19) and GL_BPaV (Supplementary Fig. S20). This QTL had reached fixation in the male sterile lines, while a small proportion of restorer lines (7.7%) had a different genotype (Fig. 5m). Interestingly, we found that this QTL had different effects on heterosis of associated traits in F1 hybrids. Being heterozygous at this locus decreased heterosis of the F1 hybrids in GYPP (average AA: 6.082 g, and average Aa: 0.890 g, Fig. 5n) and SNPP (average AA: −8.585, and average Aa: −60.920, Fig. 5o), while at the same time increasing F1 hybrid heterosis in TGW (average AA: −2.306 g, and average Aa: −1.561 g, Fig. 5p) and GL (average AA: −0.316 mm, and average Aa: −0.058 mm, Fig. 5q). These results indicate that the male sterile lines and restorer lines made different superior allele contributions to the F1 hybrids that differed among the traits.

For three-genotype QTLs, non-additive QTLs with negative effects identified in the SCA GWAS had much lower superior allele ratios (29.0% for CS and 23.4% for LS) in the male sterile lines in comparison with those of positive non-additive QTLs (44.3% for CS and 56.4% for LS) (p = 0.037 (CS) and p = 2.52 × 10^−5^ (LS), two-sided Student’s t-test, Supplementary Fig. S27). Therefore, positive non-additive QTLs (44.4% for CS and 47.4% for LS) had more female-derived superior alleles in F1 hybrids in comparison with negative non-additive QTLs (30.3% for CS and 29.0% for LS) (Supplementary Fig. S27).

### Differences in the selective pressure on male sterile lines and restorer lines contributed to differences in their QTL superior allele ratios

**Figure 6:**
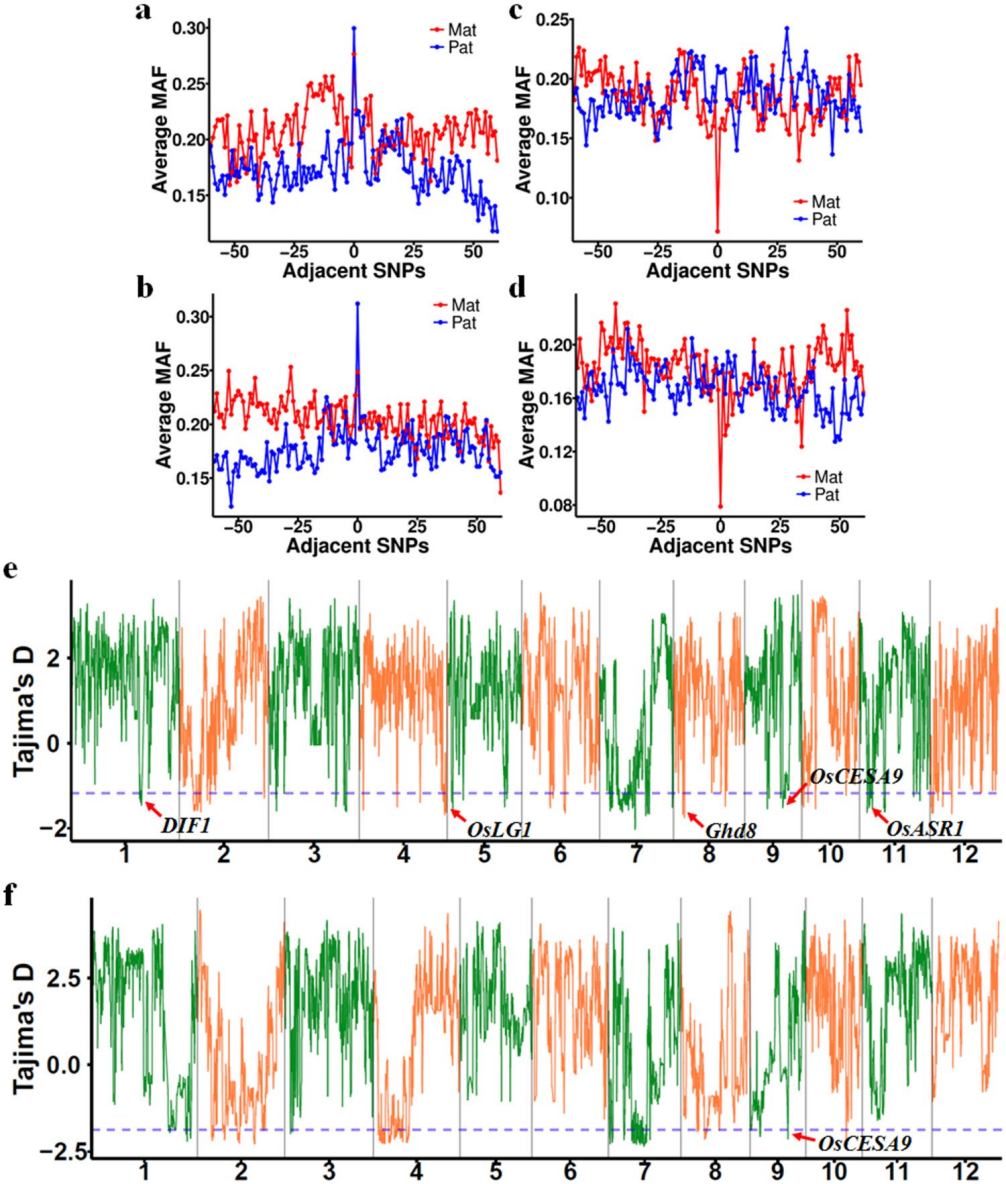
QTLs were under different selective pressure in male sterile lines and restorer lines. (**a–d**) Average MAF distributions around lead SNPs for three-genotype QTLs (a for CS, b for LS) and two-genotype QTLs (c for CS, d for LS). (**e,f**) Tajima’s D distribution across the whole genome of male sterile lines (**e**) and restorer lines (**f**). The blue dashed lines represent the 10th percentile of Tajima’s D in male sterile lines (**e**) and restorer lines (**f**).

Previous analysis showed that male sterile lines and restorer lines had different superior allele frequencies for two-genotype QTLs (Fig. 5e–h). Further analysis showed that three-genotype QTLs had much higher minor allele frequencies (MAFs) in restorer lines that those in male sterile lines (Fig. 6a and b, and Supplementary Fig. S28), while two-genotype QTLs had much lower MAFs in male sterile lines than those in restorer lines (Fig. 6c and d, and Supplementary Fig. S28). These results indicate that the selective pressure on three-genotype QTLs and two-genotype QTLs differed between the male sterile lines and restorer lines. We used Tajima’s D to measure nucleotide diversity in 240-kb sliding windows across the whole genome, and searched for overlaps between QTLs and low Tajima’s D regions (bottom 10th percentile) in the male sterile lines or restorer lines. Sixty-five (27.4%, CS) and 65 (26.1%, LS) QTLs overlapped with such regions in male sterile lines, while only 17 (7.2%, CS) and 20 (8.0%, LS) QTLs overlapped with such regions in restorer lines. Many important genes, such as *DIF*, *OsLG1*, *OsASR1*, *Ghd8*, and *OsCESA9*, were found within these regions (Fig. 6e and f). Among these QTLs, many had only two genotypes in F1 hybrids (33 (CS) and 37 (LS) for those overlapped in male sterile lines, and 12 (CS) and 16 (LS) for those overlapped in restorer lines). In addition, we found that three-genotype QTLs were located in regions with increased nucleotide diversity (high Tajima’s D) in restorer lines (Supplementary Fig. S29). These results indicate that differences in the selective pressure on male sterile lines and restorer lines contributed to differences in their superior allele frequencies.

## Discussion

Heterosis usually refers to the superior performance of F1 hybrids, such as increased growth rate and increased biomass, in comparison with their parents. However, when investigating heterosis in crop species, heterosis can vary among traits, probably because of artificial selection. For example, increased height in rice will cause lodging and thus reduce grain yield, so hybrid rice breeders favor hybrid offspring with low stature. For traits related to yield, there is no doubt that high performance in F1 hybrids is favored. In this study, we showed that F1 hybrids usually had an earlier HD, decreased SNPP and TGW, and increased PN and GYPP in comparison with their paternal parents. This variation of better paternal heterosis among traits is of practical importance as it indicates that the genetic mechanisms involved in heterosis differ among traits.

Inheritance of quantitative characteristics plays a crucial role in heterosis^12^. In this study, we showed that the original phenotypes of F1 hybrids, better paternal heterosis, and SCA, were quantitative traits (Supplementary Figs. S1–S9). These findings indicate that both better paternal heterosis and SCA are controlled by many genetic loci, consistent with the large number of QTLs identified in the BPaV GWAS and SCA GWAS (Table 1). Moreover, most loci contributing to heterosis produce minor effects on important agronomic traits in crop species subjected to intense selection^21^. For crops like corn and rice, loci with large effects on agronomic traits have been either fixed or purged during long-term artificial selection, so it is highly probable that most loci contributing to heterosis have only minor effects. This idea is consistent with our finding that large proportions of QTLs identified in the BPaV GWAS and SCA GWAS had effects much smaller than those identified in the F1 GWAS (Fig. 3d).

The BPaV and SCA GWAS results overlapped little with the F1 GWAS results, indicating that better paternal heterosis and SCA shared different genetic architectures with the original phenotypes of F1 hybrids. It is highly probable that some loci underlying better paternal heterosis and SCA do not have a significant influence on the original phenotypes of F1 hybrids. In this study, we re-ran BPaV GWAS and SCA GWAS with the addition of the original phenotypes of F1 hybrids to remove those QTLs underlying them. The results of this analysis showed that most QTLs identified in previous BPaV GWAS and SCA GWAS remained, indicating that these QTLs do not directly affect the original phenotypes of F1 hybrids. Thus, we demonstrated that both better paternal heterosis and SCA shared quite different genetic architectures with the original phenotypes of F1 hybrids. This finding was very important as it indicated that we should focus on those loci influencing heterosis directly in similar studies.

In this study, GWAS identified many QTLs related to heterosis in hybrid rice. However, the marker density was very low (approximately 0.11 SNP/kb); therefore, many important variations, including rare SNPs, copy number variation, and indels, were not identifiable. Future studies using resequencing techniques will allow most genome variations to be captured, thus revealing their relationships with heterosis. Moreover, the relatively low number of parental lines used in this study resulted in low-resolution GWAS signals. The use of many genetically diversified parental lines can greatly improve GWAS resolution and allow pseudo-overdominance and overdominance to be distinguished. Although resampling GWAS using a mixed linear model can greatly reduce false positive signals, many real associated genetic loci can be eliminated due to their relationship with population structure (see, for example, HD in rice^54, 55^). These methodological shortcomings should be remedied in future studies of rice heterosis.

Male sterile lines and restorer lines were under different levels of selective pressure and had differing demographic histories. Therefore, the genetic architectures underlying many agronomic traits could differ significantly between the male sterile lines and restorer lines. In this study, we showed that male sterile lines and restorer lines made different superior allele contributions to F1 hybrids for many QTLs due to their different superior allele ratios (Fig. 5e–h). More QTLs identified in GWAS analyses overlapped with genomic regions under selective pressure in the male sterile lines than overlapped with regions under selective pressure in the restorer lines, indicating that the male sterile lines were under much stronger selective pressure than that on the restorer lines for these agronomic traits.

Two-genotype QTLs with superior homozygotes in the F1 hybrids generally had high superior allele ratios in both parental groups (Fig. 5e and f), which indicated that these superior alleles had been under positive selection in both parental groups. In contrast, two-genotype QTLs with superior heterozygotes in the F1 hybrids usually had very low superior allele ratios in both parental groups (Fig. 5g and h). Two mechanisms might explain this phenomenon. First, these superior alleles are in strong linkage with other harmful alleles, so they are kept at low frequencies by strong negative selection pressure on the linked harmful alleles. In this situation, superior allele ratios could be increased by breaking the tight linkage between the two loci, yet this might require a large cross panel and many rounds of cross-selection. Second, these superior alleles might have been newly introduced into the hybrid rice genetic pool. In this situation, superior allele ratios could be increased though many rounds of artificial selection. These hetero-superior two-genotype QTLs might play important roles in improving agronomic traits in future hybrid rice breeding by increasing their superior allele ratios.

## Materials and Methods

### Plant materials and phenotyping

For the field experiments, we used 14 PTGMS as female parents and 3 RILs as male parents. RIL G2293 (12 lines) was derived from elite cultivars Chenghui-448 and Minghui-86. RIL G3509 (13 lines) was derived from elite cultivars Huahui-451 and 2837. RIL G4363 (14 lines) was derived from elite cultivars 2293–622 and 2464. All parental lines were used in commercial hybrid rice breeding and showed good combining ability. Each paternal line was crossed to all 14 female lines by hand pollination to derive a partial diallel cross scheme. F1 hybrids and their male parents were planted in Changsha, China, in the summer of 2014. F1 hybrids and both of their parents were planted in Lingshui, China, in the spring of 2015. All female parents were completely sterile in the high temperature summer environment of Changsha. In the low temperature spring environment of Lingshui, the female parents were partially sterile, so some panicles could be fertile depending on the temperature (threshold temperature: approximately 23.5 °C). All planting followed a randomized complete block design. Three replicates of each variety were evaluated in Changsha. Two replicates of each variety were evaluated in Linshui because of a shortage of F1 hybrid seeds. Each plot consisted of two rows with six plants per row. The distance between plants in each plot was 18.33 cm. The distance between plots was 36.66 cm. The field was managed according to normal agricultural practice. True F1 hybrids were determined by careful examination of morphological traits, including heading date, plant architecture, and grain shape. F1 hybrids with ambiguous identities were abandoned. Finally, 458 F1 hybrids from the CS dataset qualified for further phenotype evaluation, whereas 471 F1 hybrids from the LS dataset qualified for further phenotype evaluation.

Heading date was recorded per plot as the time from sowing to the day that half of all plants in that plot had emerged panicle flowers. The other traits were evaluated after harvesting, and only the middle 8 plants in each plot were harvested for further measurements. Plant height was evaluated as the length from the soil surface to the end of the main panicle. A panicle was counted if it had more than 10 full-filled grains. All grains were dried (moisture between 13% and 14%) before further evaluation of traits related to gain yield and grain shape. Approximately 600 grains were used to measure grain shape-related traits (TGW, GL, GW, and GLWR). SNPP was measured using the following formula: $\mathrm{SNPP}=\mathrm{GYPP}/(\mathrm{PN}\,\ast \,\mathrm{TGW})$.

### Genotyping and imputation

All 53 parental lines were genotyped using the rice 50 K SNP chip on the Illumina Infinium platform. This SNP array contains 51478 SNPs that are evenly distributed on all 12 chromosomes. First, we filtered the genotypes of the parental lines by the following criteria: missing rate <= 0.2 (4466 SNPs removed) and heterozygous rate <= 0.15 (another 1596 SNPs removed). The filtering process left 45416 high quality SNPs, among which 26920 SNPs showed polymorphism and were used for further analysis. We used the KNNcatimpute function (R package ‘scrime’) to perform genotype imputation. To increase imputation accuracy, we added the high-quality genotypes of 337 closely related rice cultivars to form an imputation panel consisting of 390 samples. We evaluated the imputation accuracy by random sub-sampling validation, and the accuracy rate was ~98.5%. After imputation, we obtained F1 hybrid genotypes by combining their corresponding parental genotypes. The F1 hybrid genotypes were filtered to leave only those with MAF >= 0.05, leaving 26736 SNPs for further analysis.

### Phylogenetic analysis and population structure

We performed phylogenetic analysis for all parental lines using a neighbor-joining statistical method, whereas a bootstrap method was used to test for phylogeny (200 bootstraps). Phylogenetic analysis was carried out using MEGA software (version: 6.06)^56^. Population structure analysis was carried out for F1 hybrid genotypes using smartpca software (EIGENSOFT software package, version: 6.0.1)^57^.

### Phenotype normalization and resample GWAS

Mixed linear models have been used in GWAS to control for population structure and familial relationships. One important assumption for mixed linear models is that the phenotype residues have a Gaussian distribution; any deviation from this assumption may cause spurious signals and reduce power^58^. We found that the phenotype distributions of some traits in our phenotype datasets were highly skewed or bimodal. Therefore, python package WarpedLMM (version: 0.21)^59^ was used to perform phenotype normalization to ensure a Gaussian distribution of the phenotype residues before further GWAS analysis.

To obtain a deep understanding of heterosis in hybrid rice, we separated each trait into three categories, F1, BPaV, and SCA, and identified genetic mechanisms underlying these trait categories by GWAS analysis. F1 represents the original phenotype values of the F1 hybrids. BPaV represents the better paternal values of the F1 hybrids ($\mathrm{BPaV}={\rm{F}}1-\mathrm{Pat}$). SCA represents the special combining abilities of the F1 hybrids ($\mathrm{SCA}\,=\,{\bar{y}}_{{ij}}-{\bar{y}}_{..}-{{GCA}}_{{mat}}-$ ${{GCA}}_{{pat}}$, and ${\mathrm{GCA}}_{{\rm{i}}}={\bar{y}}_{i.}-{\bar{y}}_{..}$; ${\bar{y}}_{{ij}}$ represents the average phenotype of F1 hybrids that derived from parents i and j, ${\bar{y}}_{..}$ represents the overall mean of all crosses, ${\bar{y}}_{i.}$ represents the average value of all F1’s that derived from parent i, and GCA represents general combining ability). We also performed MPV GWAS for some traits in the LS dataset, with MPV defined as the non-additive performance of the F1 hybrid compared to the mean of both parents (${\rm{MPV}}={\rm{F}}1-({\rm{Pat}}+{\rm{Mat}})/2$). Our study panel was highly structured, and many F1 hybrids had close familial relationships, leading to two problems: spurious association signals and long range correlations among genetic markers that made it difficult to interpret the association analysis results^38, 40^. To solve the first problem, we used a mixed linear model to control for population structure and close relationships among F1 hybrids. To solve the second problem, we used the forward-selection resampling GWAS method (based on a multiple loci model) instead of the single locus model used in traditional GWAS^38, 40, 41^. First, we randomly selected 80% of all samples from the total dataset without replacement to form a new sub-dataset. This selection procedure was repeated 300 times to form 300 sub-datasets. GWAS using a mixed linear model was performed iteratively for each sub-dataset, after which the SNP with the lowest P-value, provided that it passed the significance threshold, was added as a covariate for the next round of GWAS. This forward-selection GWAS procedure was repeated until no additional SNP passed the significance threshold. After finishing the GWAS analysis for all sub-datasets and averaging the results across all identified genetic models, a resample model inclusion probability (RMIP, ranging from 1 to 100), representing the model inclusion probability in 100 forward-selection resampling GWAS analyses, was assigned to each identified SNP. Those SNPs meeting the empirical RMIP threshold of 5 were identified as significantly correlated SNPs. GWAS analysis was performed using all 4 inheritance models: additive, dominance, recessive, and over-dominance. The additive inheritance model was utilized as in traditional GWAS (AA, Aa, and aa were coded as 0, 1, and 2, respectively). For dominance and recessive inheritance models, heterozygotes (Aa) were coded the same as either the homozygous genotypes of major alleles (AA) or homozygous genotypes of minor alleles (aa). The over-dominance model was utilized by recoding the heterozygote to 1 and both homozygotes to 0. Among the four RMIPs derived from these inheritance models, the biggest one was used for further analysis. To reduce the redundancy of association signals, we merged SNPs located less than 800 kb apart and showed correlations by linear regression analysis. GWAS was performed using R package ‘GenABEL’ (mmscore function)^60, 61^. The genome-wide significance threshold for each GWAS analysis was determined by 300 permutation tests at a false discovery rate (FDR) lower than 0.05 using the Benjamini and Hochberg method^62^.

To define the QTL range, we first split the parent genomes into hapblocks using the software HAPLOVIEW^63^ and the recombinant confidence interval method devised by Gabriel *et al*.^64^. Then the QTL regions were determined by the range of the corresponding hapblocks. All genomic positions provided in this manuscript are based the rice reference genome IRGSP-Build4.

### Variance explained

The variance explained by each QTL was estimated as the sum of squares by a linear regression model using the “lm” function in R. For the variance explained by multiple QTLs, we first filtered the QTLs using a forward-backward selection method based on the Akaike information criterion (AIC), after which the total variance explained was estimated as the residual sum of squares by fitting all filtered QTLs after removal of family effects (variance explained by the top 10 genomic PCs).

### Assessment of QTL effects in F1 hybrids

For each QTL identified in the GWAS analysis, we separated all F1 hybrids into three groups according to their genotypes (one heterozygote (Aa) or two homozygotes (AA, and aa)). If any of these three groups had less than 15 samples, the QTL was placed into the two-genotype group; otherwise, it was placed into the three-genotype group. For two-genotype QTLs, we directly compared the average phenotype values of both two genotype classes. A two-genotype QTL was defined as hetero-superior if the average performance of its heterozygotes outperformed that of its homozygotes in the F1 hybrids; otherwise, it was defined as homo-superior. For three-genotype QTLs, we used a linear regression model to assess dominance/additive (d/a) effects by Plink software (–linear, –genotypic, version: 1.9)^65^. Dominance effects were assessed by recoding the three genotypes (AA, Aa, and aa as 0, 1, and 0, respectively), whereas additive effects were assessed by recoding the three genotypes as 0, 1, and 2. Dominance and additive effects were fitted simultaneously in the linear regression model, and the top 10 genomic PCs were added as covariates to control for population structure. We used the following d/a criteria judged by Stuber *et al*.^66^ to define the QTL effects: additive = [0, 0.2); partial dominance = [0.2, 0.8]; dominance = (0.8, 1.2]; overdominance >1.2.

### Selective scan in parental lines

We used Tajima’s D to find genomic regions under selective pressure in the 14 male sterile lines and 39 restorer lines, respectively. To include enough polymorphic sites for robust analysis results, we used a 240-kb sliding window size (containing approximately 15 polymorphic sites) with a 20-kb step size. A QTL was defined to be under selective pressure if it overlapped with any genomic region below the 10th percentile of Tajima’s D in either male sterile lines or restorer lines. Calculation of Tajima’s D was performed using Variscan software (version 2.0)^67^.

## Electronic supplementary material

Supplemental File

Dataset 1

Dataset 2

Dataset 3
