# Supplementary material for: Genome-wide dissection of heterosis for yield traits in two-line hybrid rice populations
Source: Sci Rep. 2017 Aug 9;7:7635. doi: 10.1038/s41598-017-06742-7 (PMC5550440; doi:10.1038/s41598-017-06742-7)

Genome-wide dissection of heterosis for yield traits in two-line hybrid rice populations

Gang Zhen[1], Peng Qin[2], Kai Yu Liu[2], Dong Yang Nie[2],

Yuan Zhu Yang[2*], Xing Wang Deng[1*], Hang He[1*]

[1] State Key Laboratory of Protein and Plant Gene Research, Peking-Tsinghua Center for Life Sciences, School of Advanced Agriculture Sciences and School of Life Sciences, Peking University, Beijing, China.

[2] Ava Seed Academy of Sciences, Changsha, Hunan, China.

[*] Corresponding authors

Email:

zhengang@pku.edu.cn (GZ)

576276568@qq.com (PQ)

328471977@qq.com (KYL)

243553471@qq.com (DYN)

hehang@pku.edu.cn (HH)

deng@pku.edu.cn (XWD)

yzhuyah@163.com (YZY)

Supplementary Data S1: Genotypes of all parental lines.

Supplementary Data S2: Phenotypes of all F1 hybrids and male parents.

Supplementary Data S3: All QTLs identified in GWAS analysis. Genomic positions in this dataset are based on rice reference genome IRGSP-Build4.

Supplementary Figure S1: Phenotype distributions of heading date in three F1 hybrid groups. First panel (a,b): original phenotype value. Second panel (c,d): better paternal value. Third panel (e,f): special combining ability. Fourth panel (g): middle parent value. The left panel (a,c,e) was measured in Changsha, and the right panel (b,d,f,g) was measured in Lingshui.

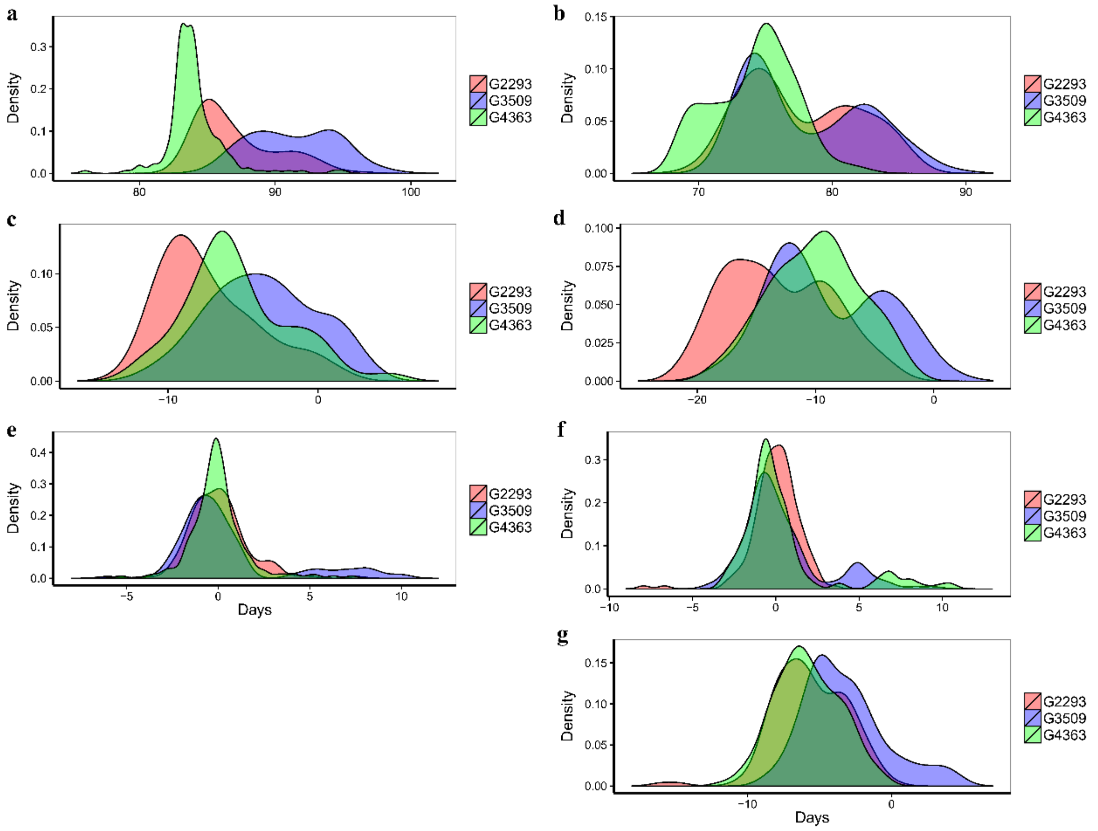

Supplementary Figure S2: Phenotype distributions of plant height in three F1 hybrid groups. First panel (a,b): original phenotype value. Second panel (c,d): better paternal value. Third panel (e,f): special combining ability. Fourth panel (g): middle parent value. The left panel (a,c,e) was measured in Changsha, and the right panel (b,d,f,g) was measured in Lingshui.

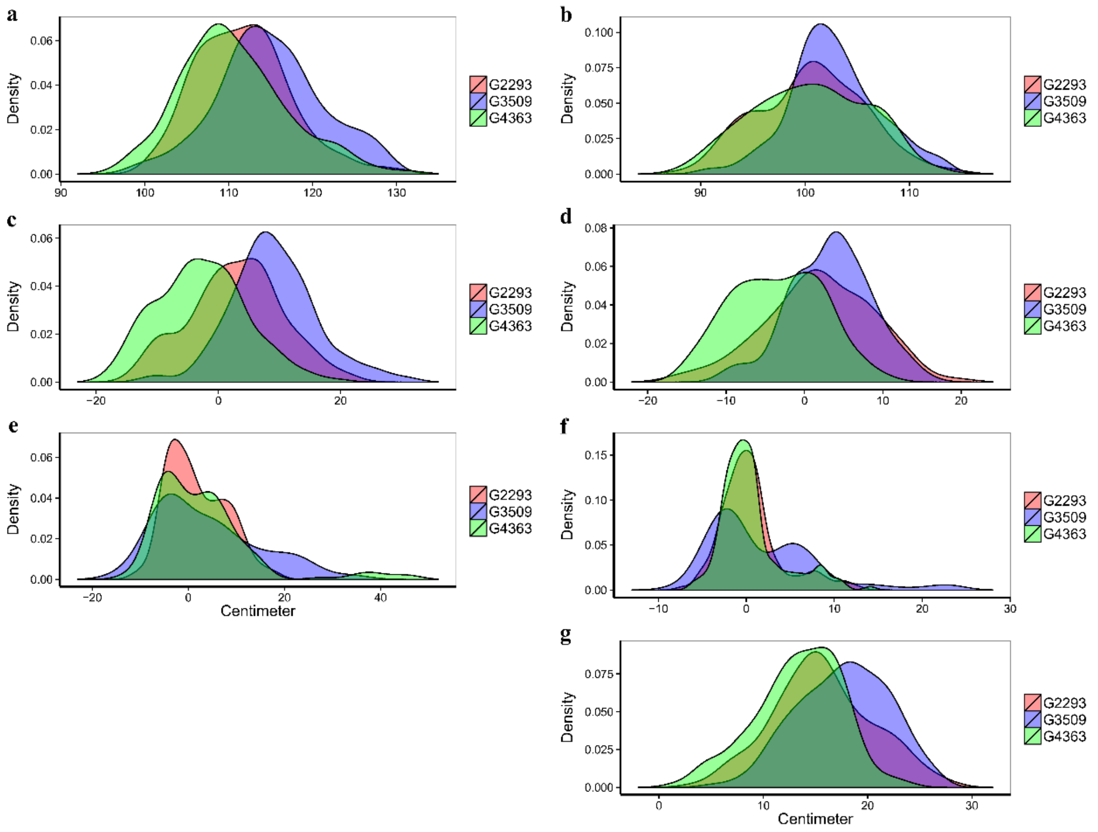

Supplementary Figure S3: Phenotype distributions of panicle number in three F1 hybrid groups. First panel (a,b): original phenotype value. Second panel (c,d): better paternal value. Third panel (e,f): special combining ability. The left panel (a,c,e) was measured in Changsha, and the right panel (b,d,f) was measured in Lingshui.

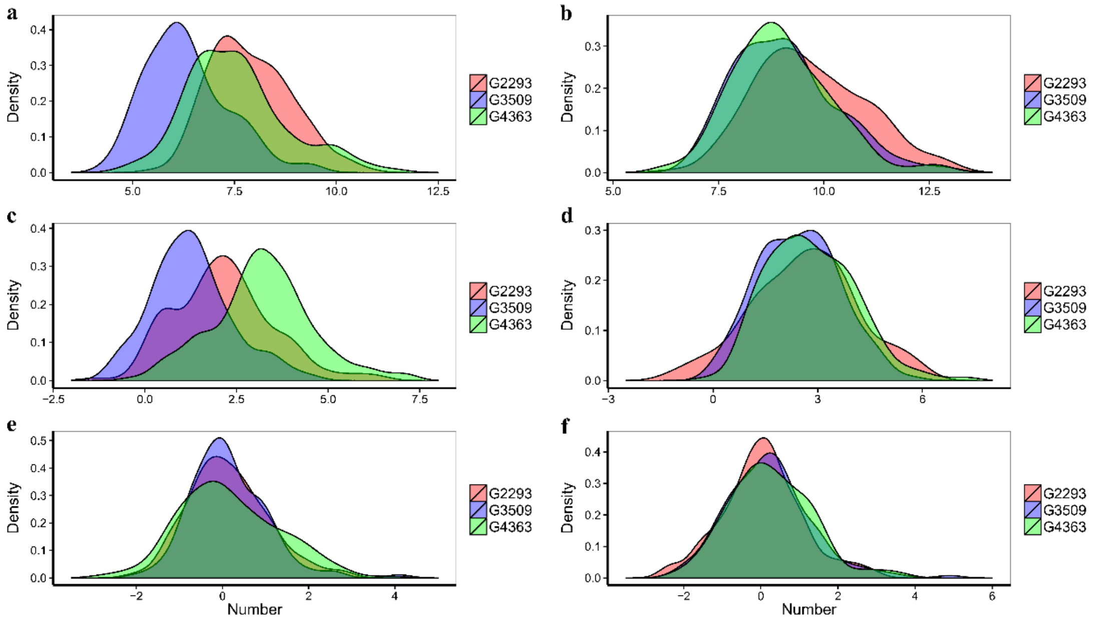

Supplementary Figure S4: Phenotype distributions of seeds number per panicle in three F1 hybrid groups. First panel (a,b): original phenotype value. Second panel (c,d): better paternal value. Third panel (e,f): special combining ability. The left panel (a,c,e) was measured in Changsha, and the right panel (b,d,f) was measured in Lingshui.

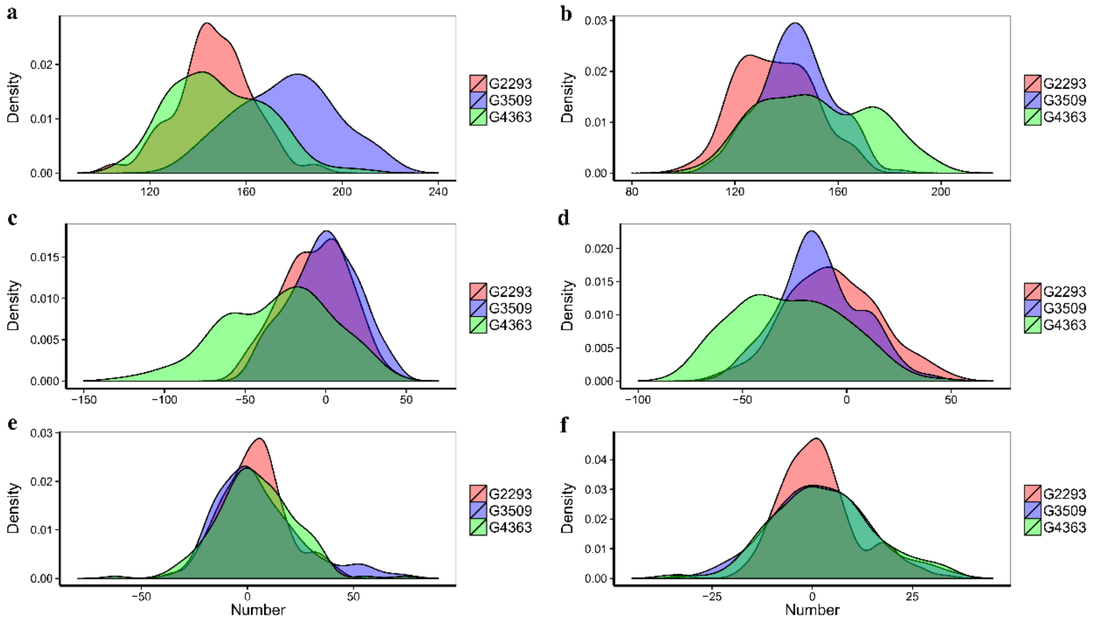

Supplementary Figure S5: Phenotype distributions of grain yield per plant in three F1 hybrid groups. First panel (a,b): original phenotype value. Second panel (c,d): better paternal value. Third panel (e,f): special combining ability. The left panel (a,c,e) was measured in Changsha, and the right panel (b,d,f) was measured in Lingshui.

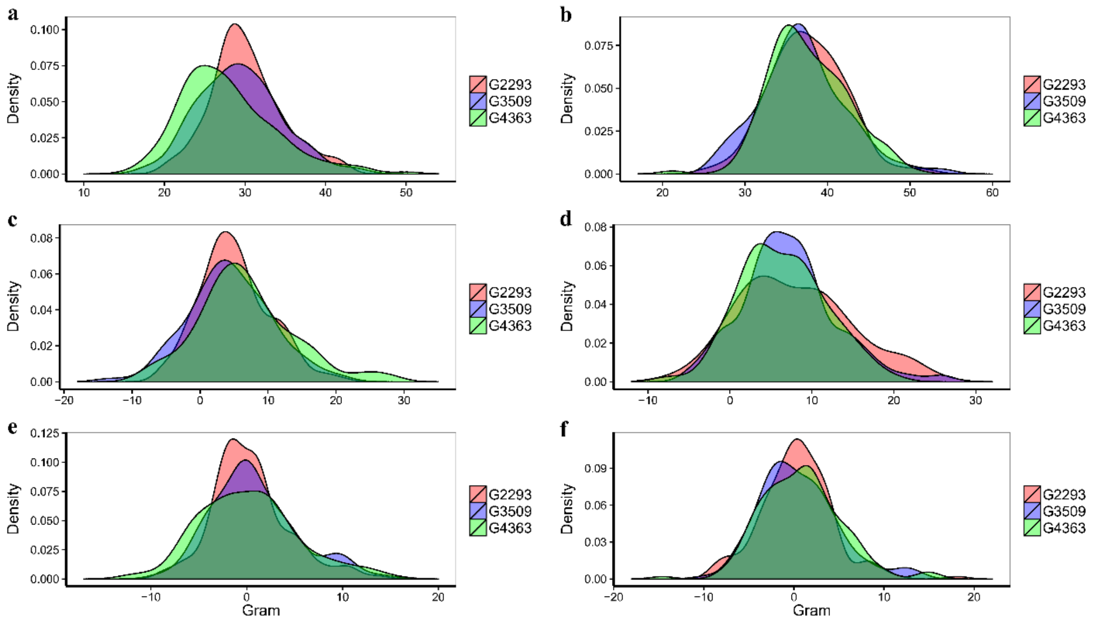

Supplementary Figure S6: Phenotype distributions of 1000 grain weight in three F1 hybrid groups. First panel (a,b): original phenotype value. Second panel (c,d): high paternal value. Third panel (e,f): special combining ability. Fourth panel (g): middle parent value. The left panel (a,c,e) was measured in Changsha, and the right panel (b,d,f,g) was measured in Lingshui.

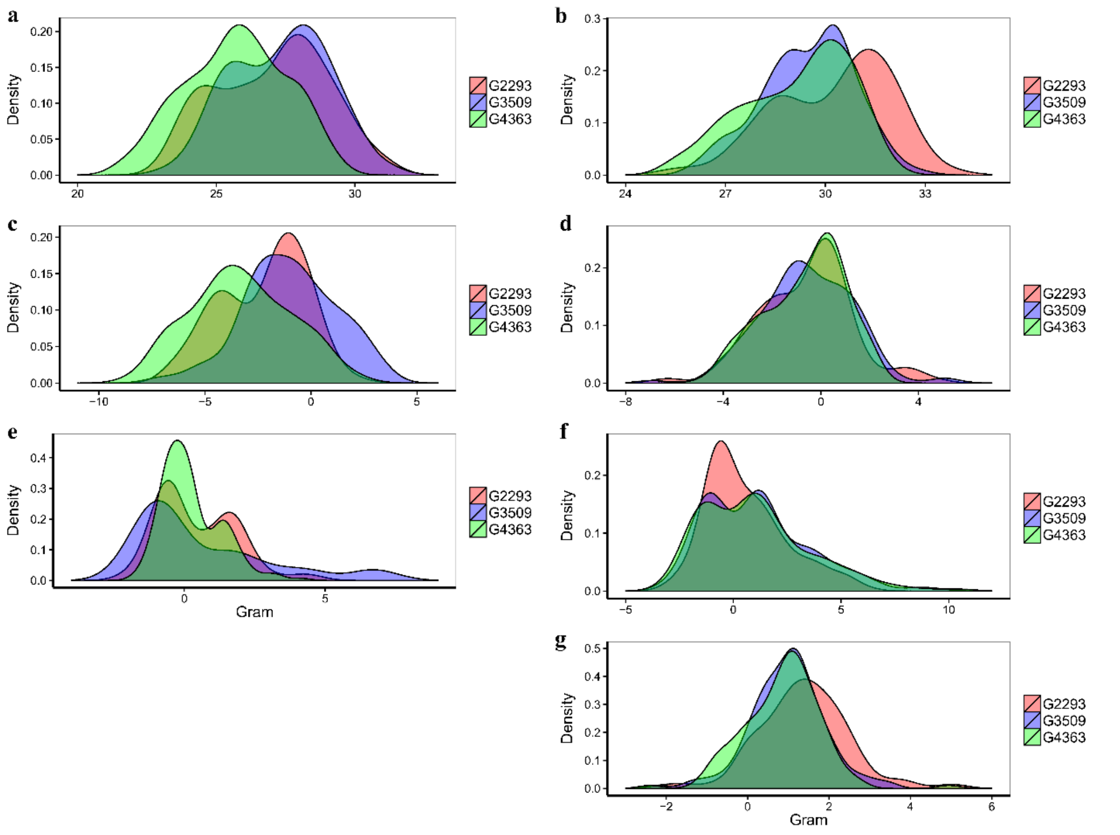

Supplementary Figure S7: Phenotype distributions of grain length in three F1 hybrid groups. First panel (a,b): original phenotype value. Second panel (c,d): better paternal value. Third panel (e,f): special combining ability. Fourth panel (g): middle parent value. The left panel (a,c,e) was measured in Changsha, and the right panel (b,d,f,g) was measured in Lingshui.

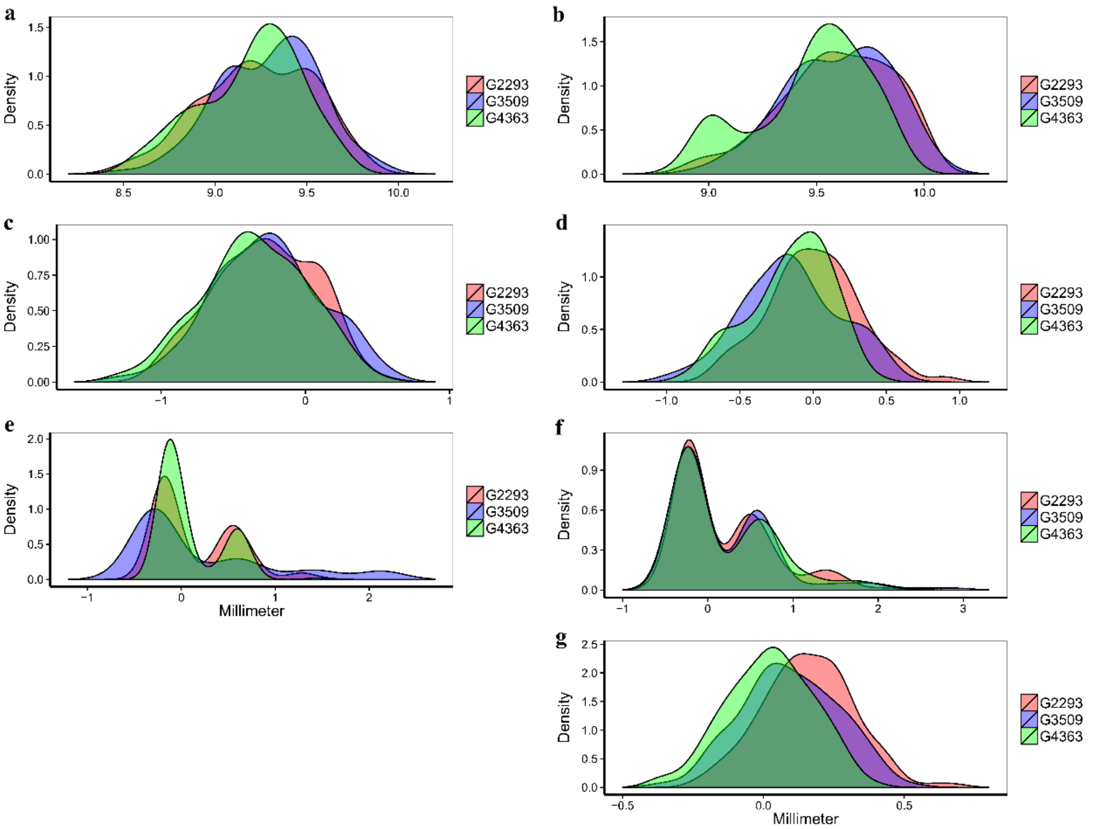

Supplementary Figure S8: Phenotype distributions of grain width in three F1 hybrid groups. First panel (a,b): original phenotype value. Second panel (c,d): better paternal value. Third panel (e,f): special combining ability. Fourth panel (g): middle parent value. The left panel (a,c,e) was measured in Changsha, and the right panel (b,d,f,g) was measured in Lingshui.

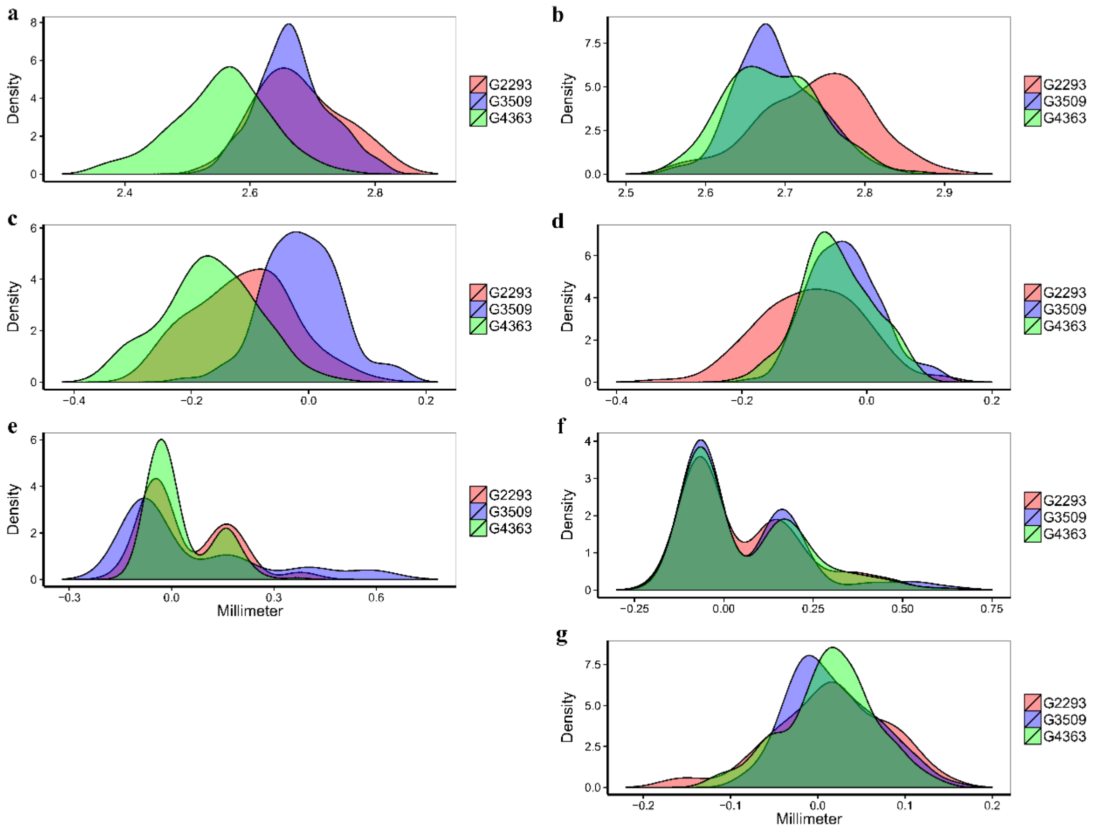

Supplementary Figure S9: Phenotype distributions of grain length/width ratio in three F1 hybrid groups. First panel (a,b): original phenotype value. Second panel (c,d): better paternal value. Third panel (e,f): special combining ability. Fourth panel (g): middle parent value. The left panel (a,c,e) was measured in Changsha, and the right panel (b,d,f,g) was measured in Lingshui.

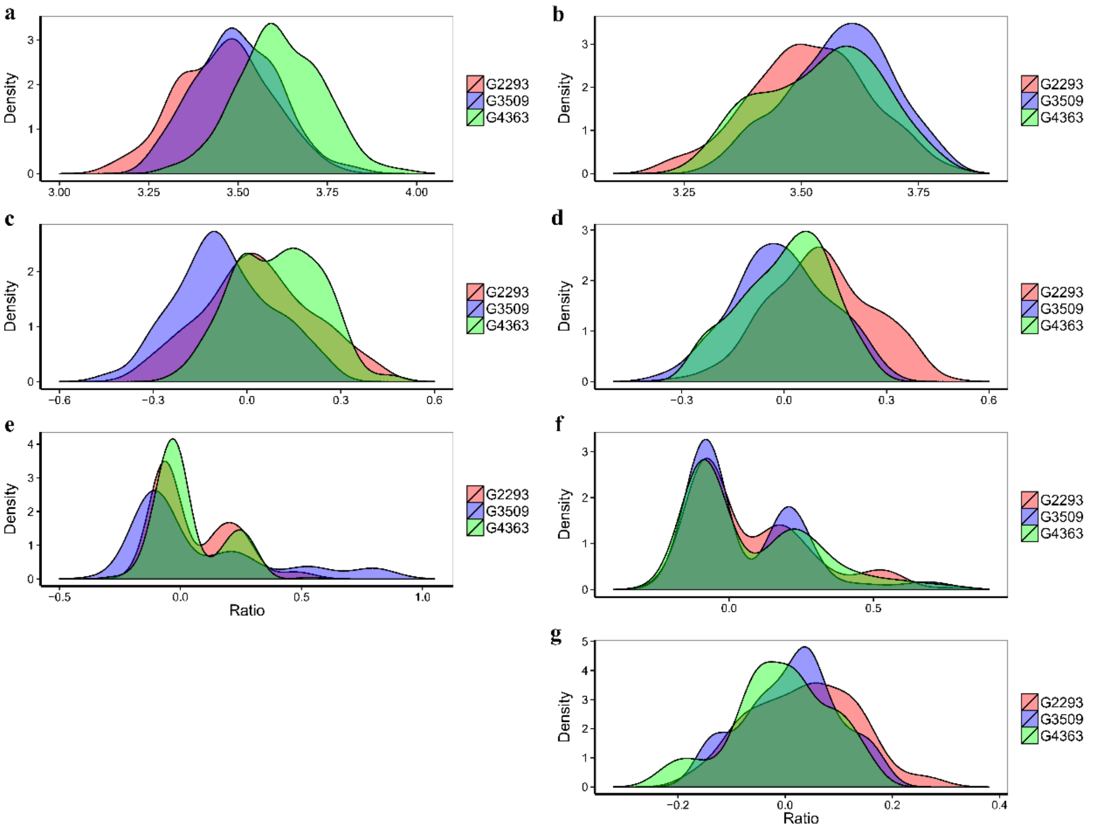

Supplementary Figure S10: Phenotype comparisons of nine agronomic traits between F1 hybrids and their male parents. (a) Heading date; (b) Plant height; (c) Panicle number; (d) Seeds number per panicle; (e) Grain yield per plant; (f) 1000 grain weight; (g) Grain length; (h) Grain width; (i) Grain length/width ratio. The black dashed lines represent equalities between F1 hybrids and their male parents.

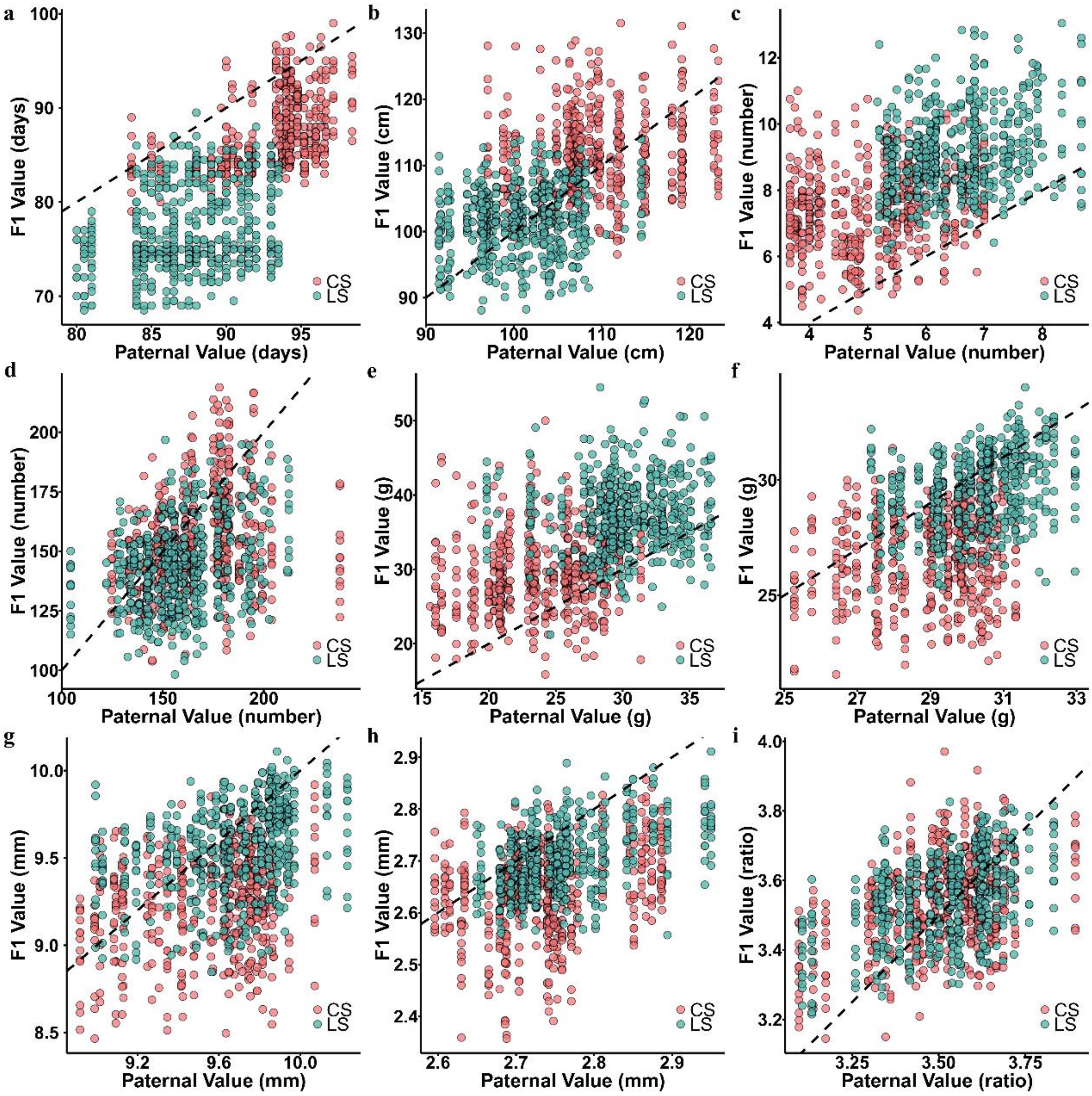

Supplementary Figure S11: Phenotype comparisons of six agronomic traits between F1 hybrids and middle parent values in the LS dataset. (a) Heading date; (b) Plant height; (c) 1000 grain weight; (d) Grain length; (e) Grain width; (f) Grain length/width ratio. The black dashed lines represent equalities between F1 hybrids and middle parent values.

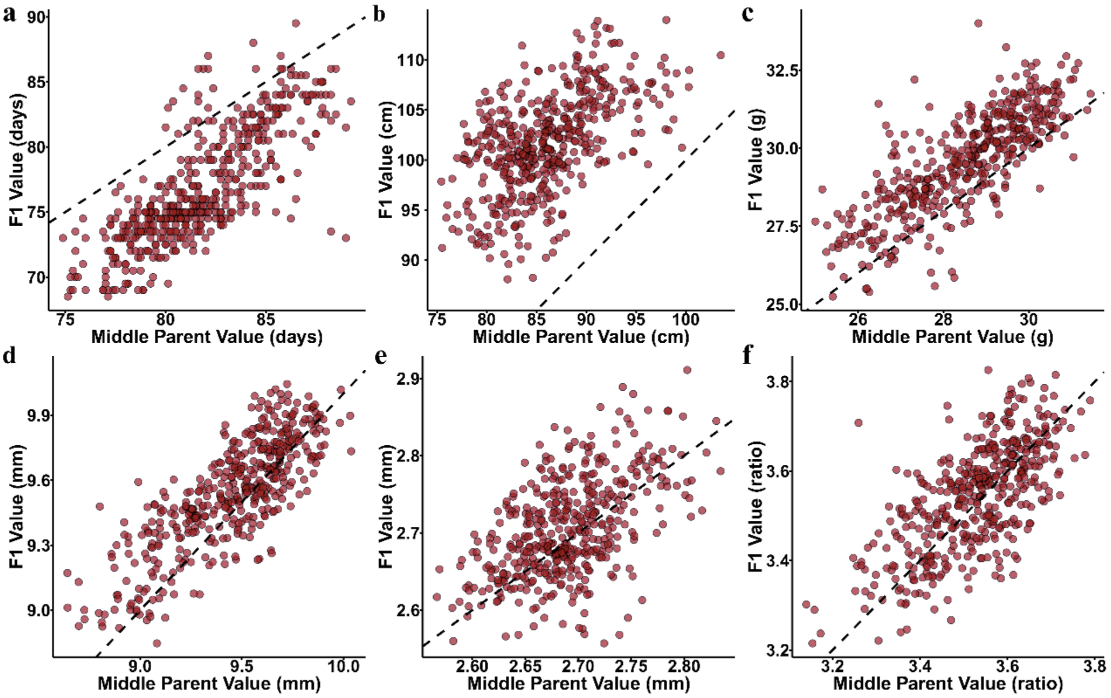

Supplementary Figure S12: Phenotypic correlations among different traits. Correlations were measured in Pearson correlation coefficients (r value). The left bottom panel was phenotype correlations in the CS dataset, and the right top panel was phenotype correlations in the LS dataset.

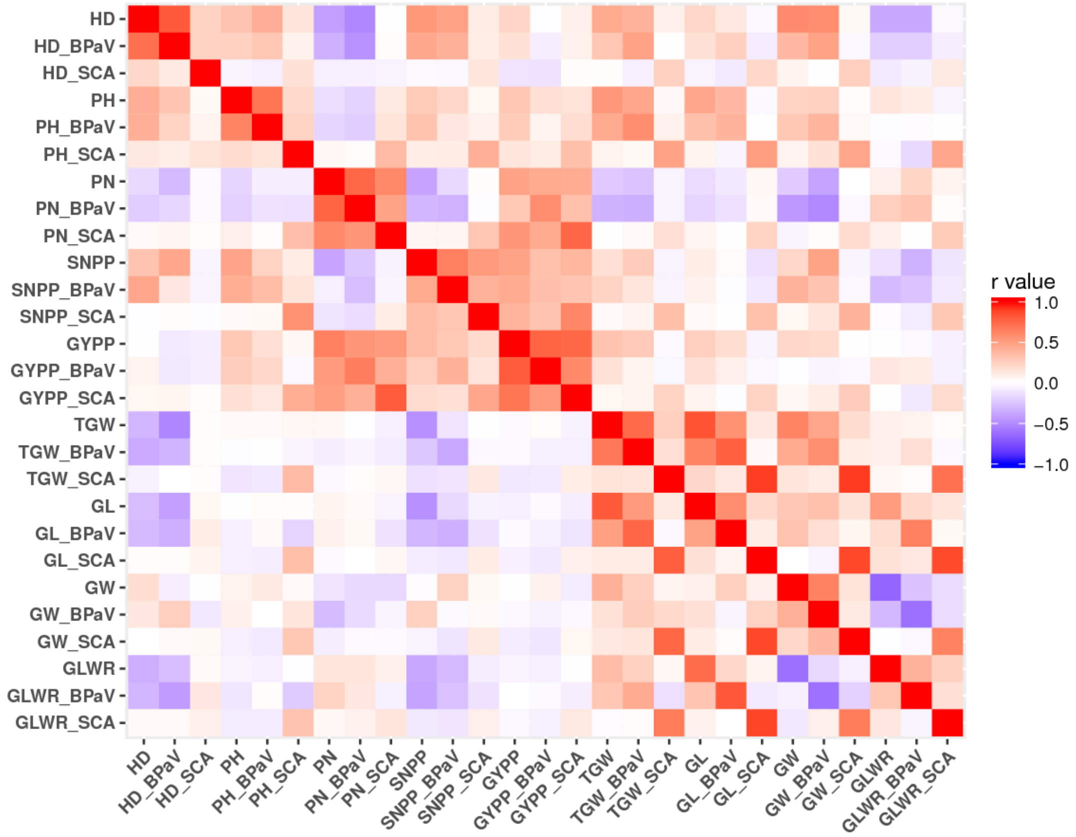

Supplementary Figure S13: Contributions of four yield-related traits (HD, PN, SNPP, and TGW) to GYPP total variance.

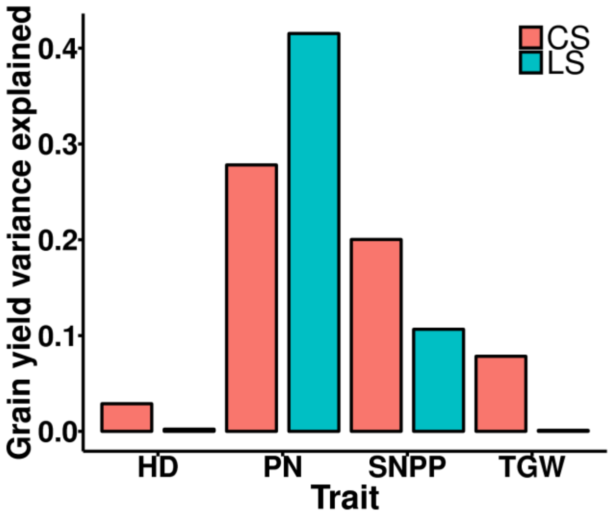

Supplementary Figure S14: F1 hybrids' whole genome heterozygosity distribution (a), and average heterozygosity per 100-kb across the whole genome of F1 hybrids (b). The red dashed lines in (b) represent the positions of centromeres.

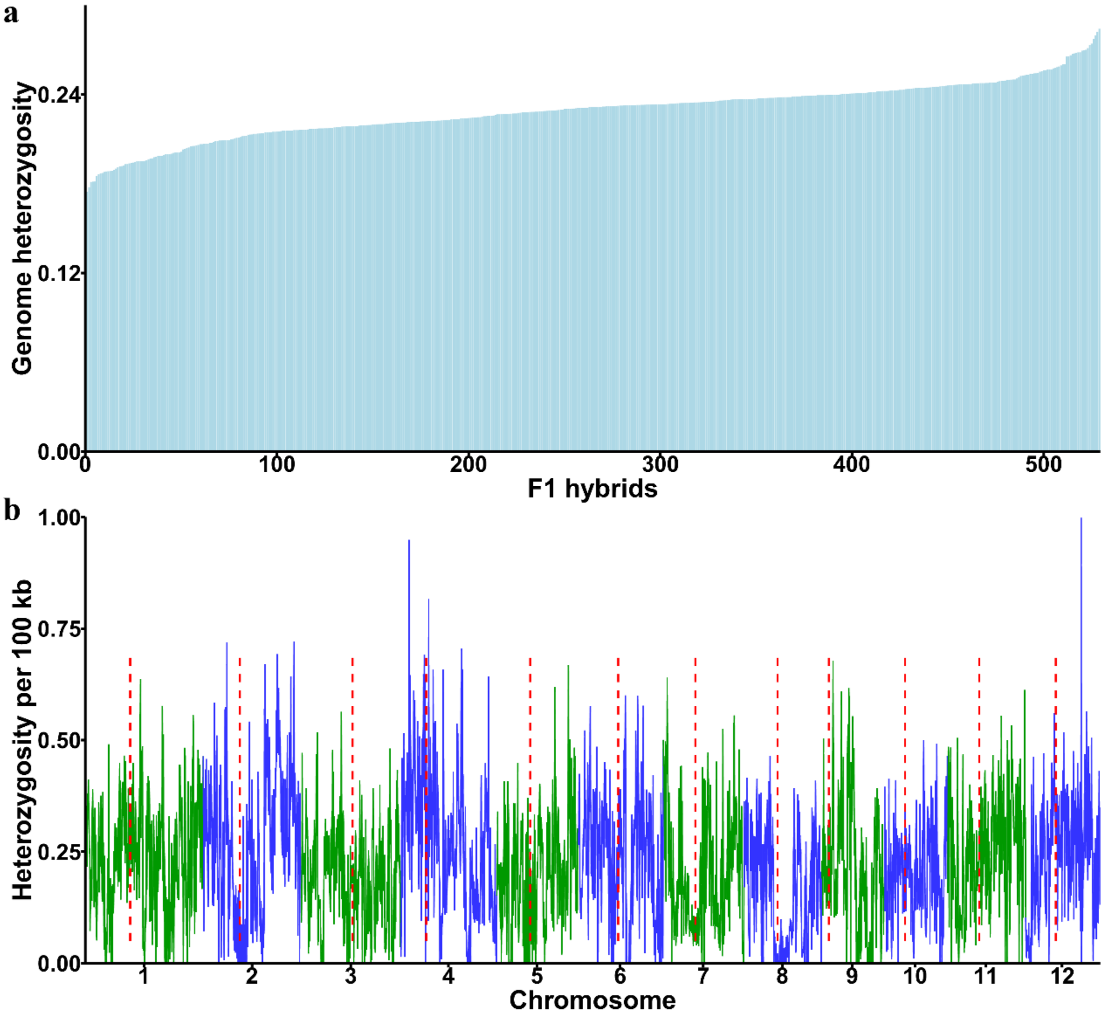

Supplementary Figure S15: Frequency distributions of RMIP in F1 GWAS (a), BPaV GWAS (b), and SCA GWAS (c) results.

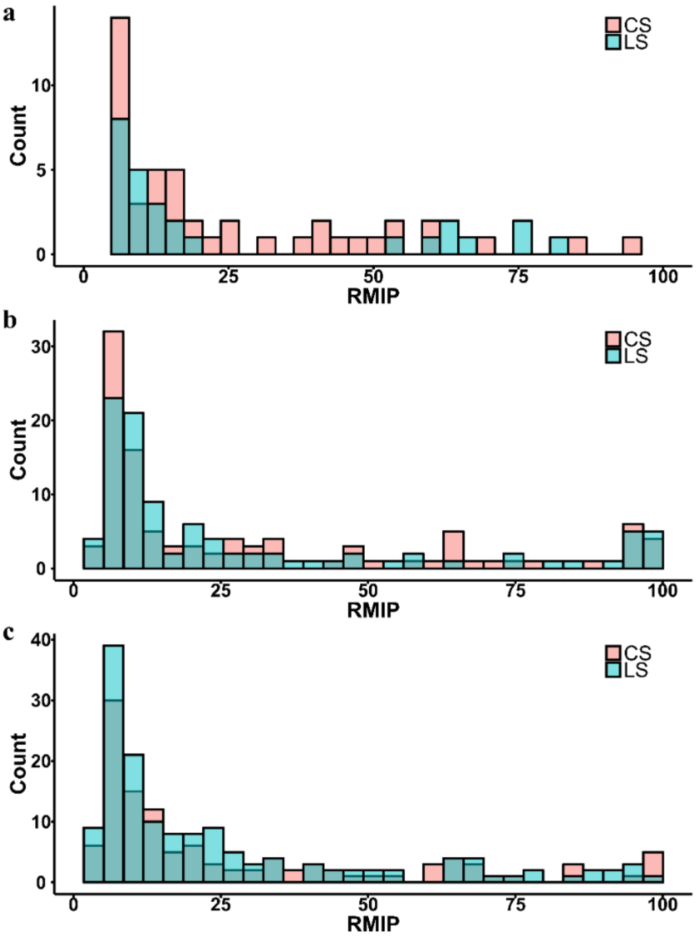

Supplementary Figure S16: Manhattan plots of PH GWAS results for the CS dataset (a) and LS dataset (b).

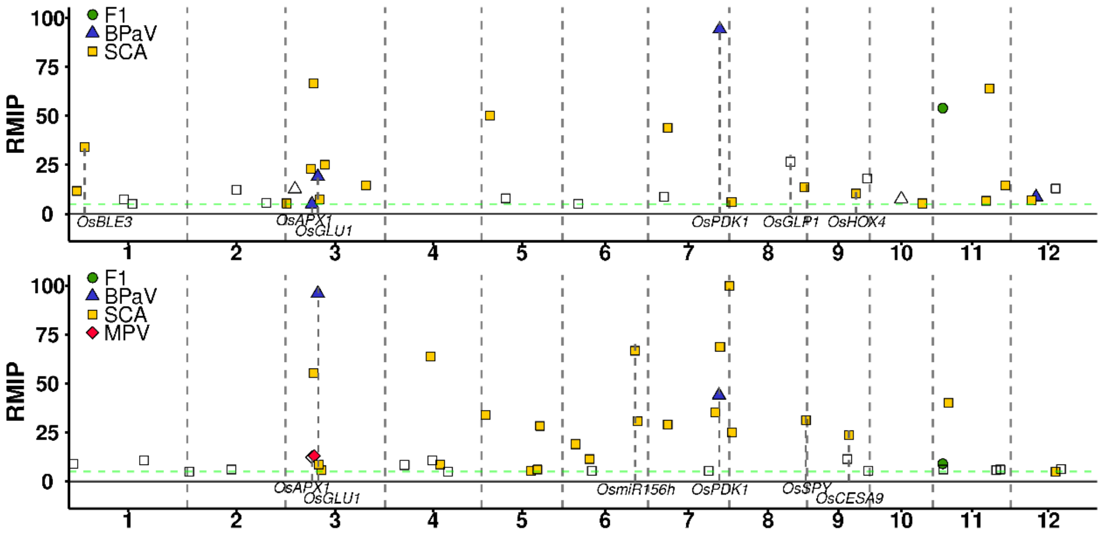

Supplementary Figure S17: Manhattan plots of PN GWAS results for the CS dataset (a) and LS dataset (b).

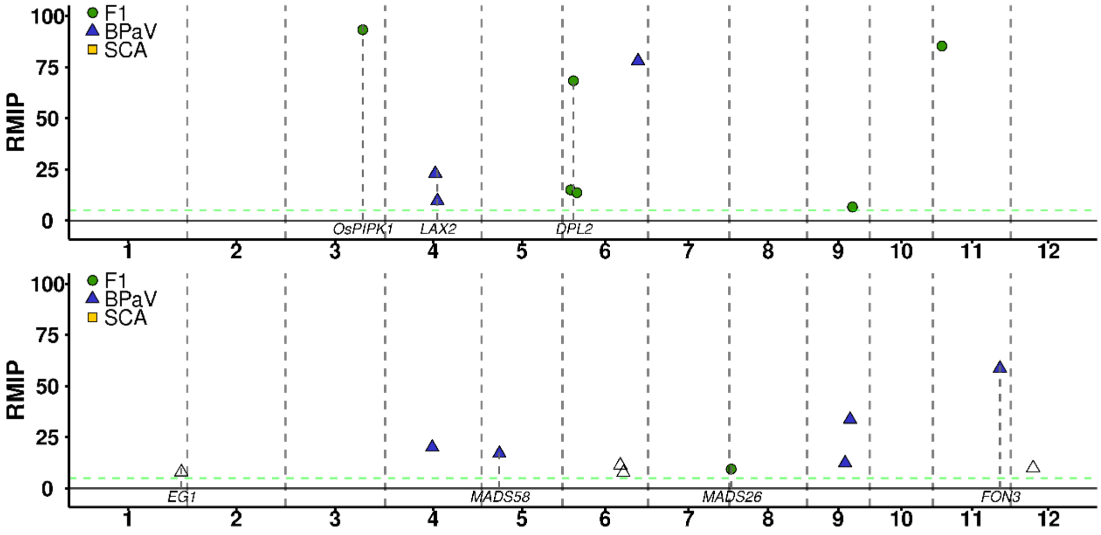

Supplementary Figure S18: Manhattan plots of GYPP GWAS results for the CS dataset (a) and LS dataset (b).

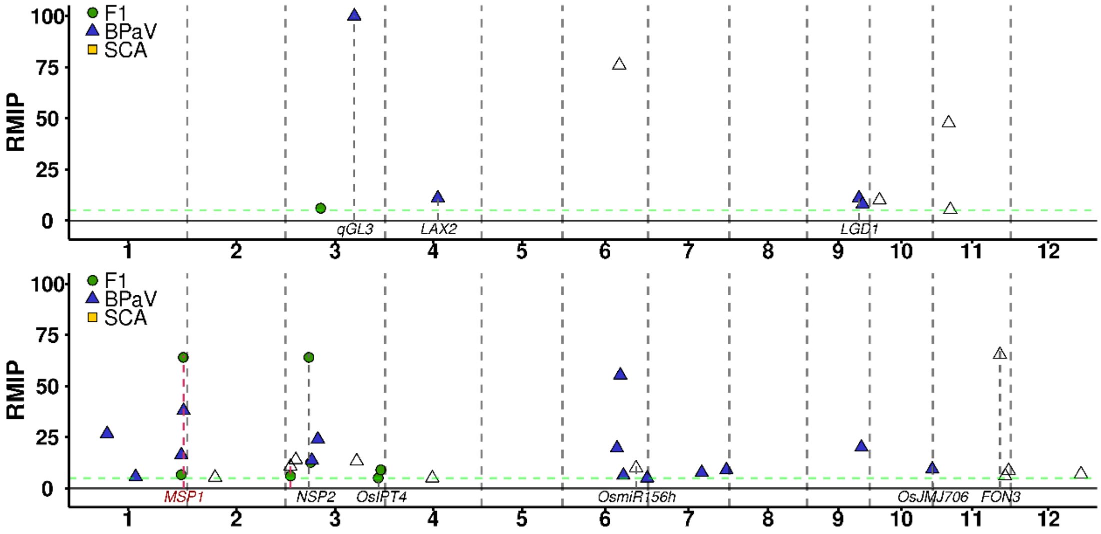

Supplementary Figure S19: Manhattan plots of TGW GWAS results for the CS dataset (a) and LS dataset (b).

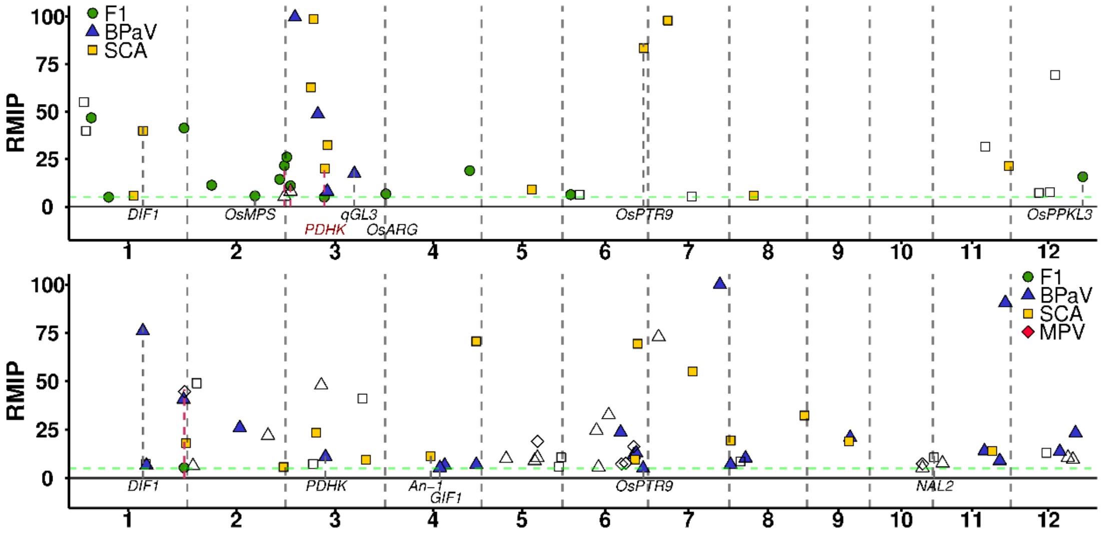

Supplementary Figure S20: Manhattan plots of GL GWAS results for the CS dataset (a) and LS dataset (b).

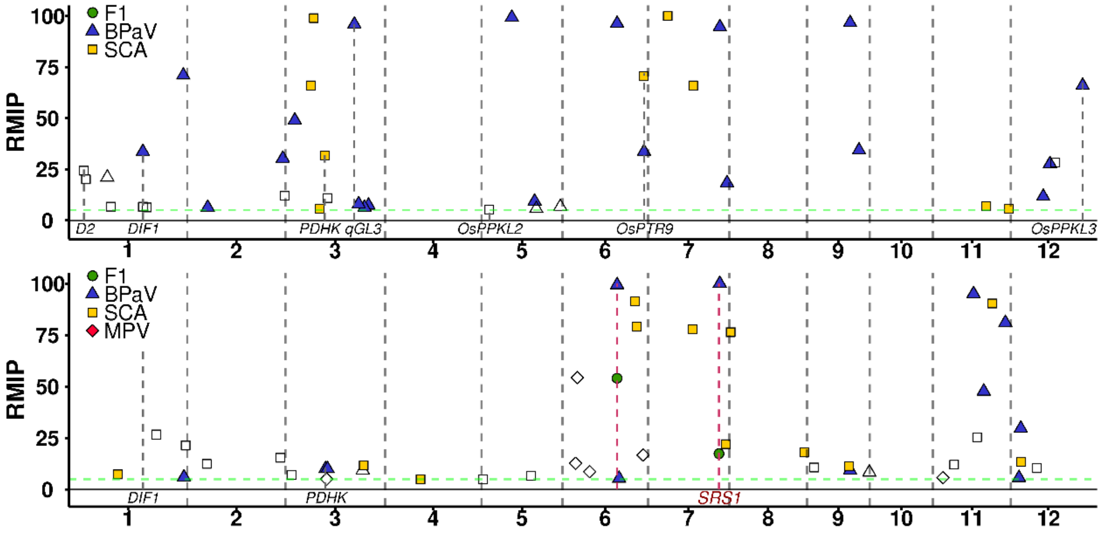

Supplementary Figure S21: Manhattan plots of GW GWAS results for the CS dataset (a) and LS dataset (b).

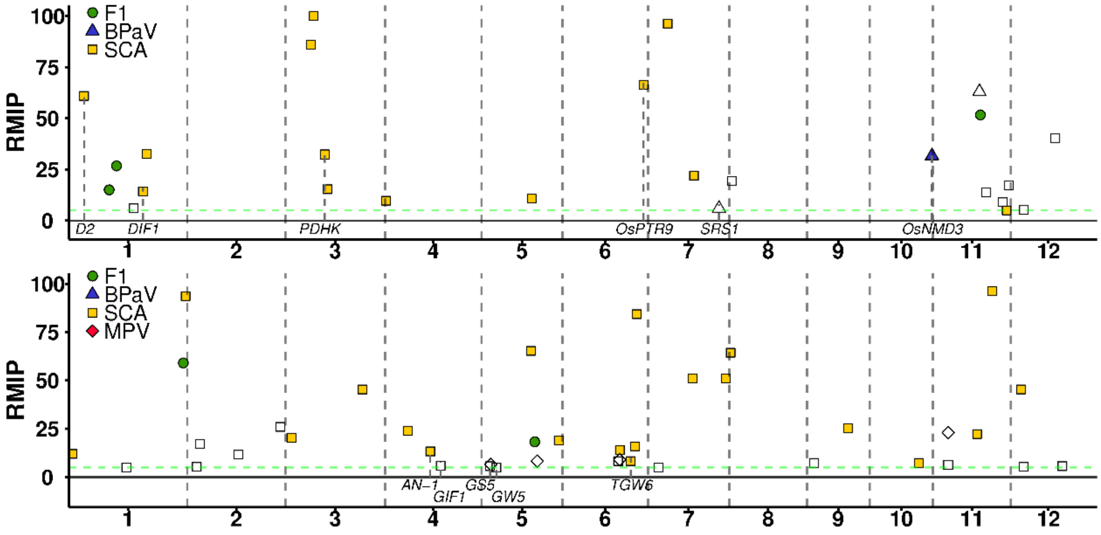

Supplementary Figure S22: Manhattan plots of GLWR GWAS results for the CS dataset (a) and LS dataset (b).

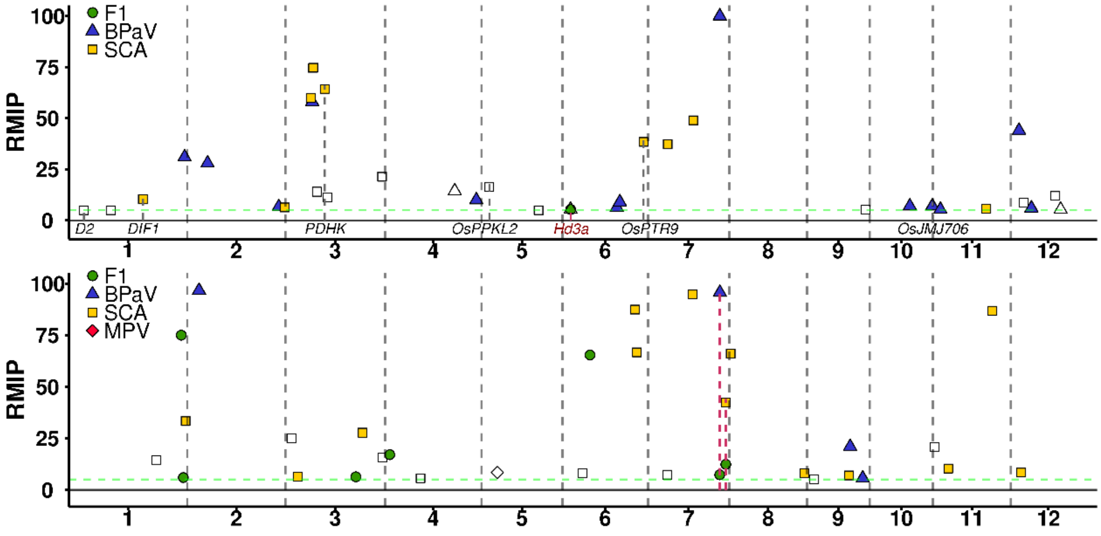

Supplementary Figure S23: Boxplots showing RMIP distributions of QTLs remained or wiped out in new GWAS analysis.

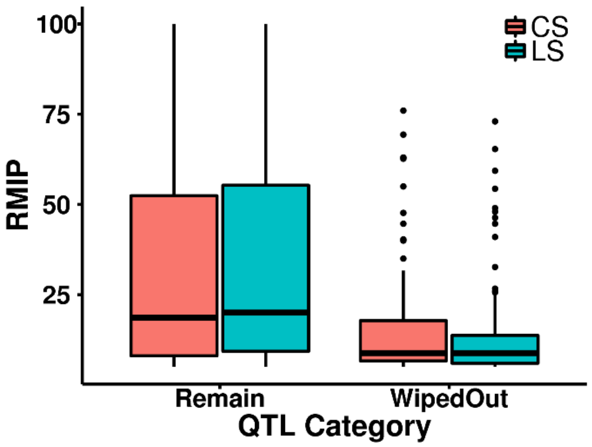

Supplementary Figure 24: Proportions of QTLs that showed additive (Add), partial-dominance (Partial-Dom), dominance (Dom), and overdominance (OverDom). (a) QTLs identified in CS; (b) QTLs identified in LS.

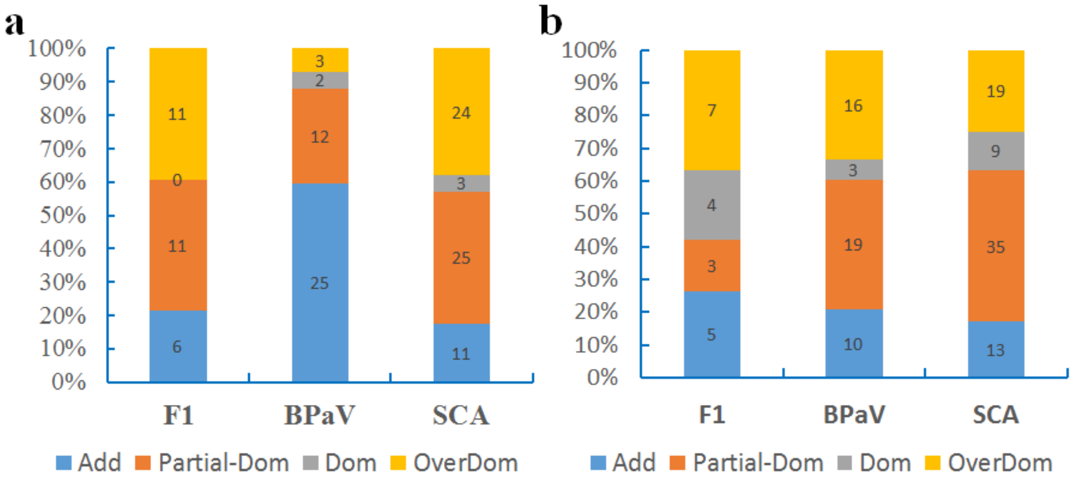

Supplementary Figure S25: Phylogenetic relationships among 14 male sterile lines (red thick branches), 3 RILs (blue thick branches), additional 36 male sterile lines (red thin branches), and additional 48 restorer lines (blue thin branches).

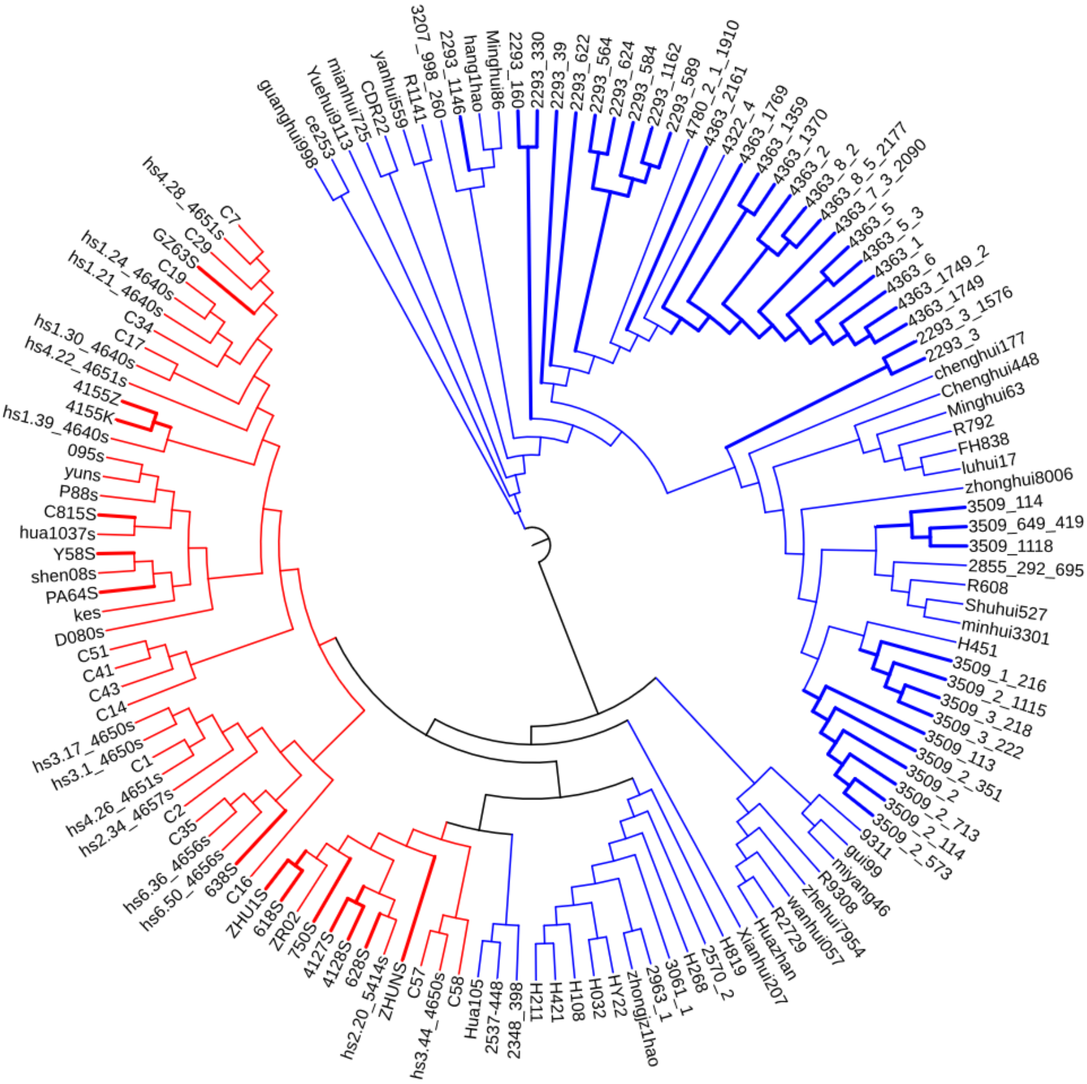

Supplementary Figure S26: Superior allele ratios of different two-genotype QTL groups in additional 36 male sterile lines (red) and 48 restorer lines (light blue). (a) HPaV GWAS results; (b) SCA GWAS results.

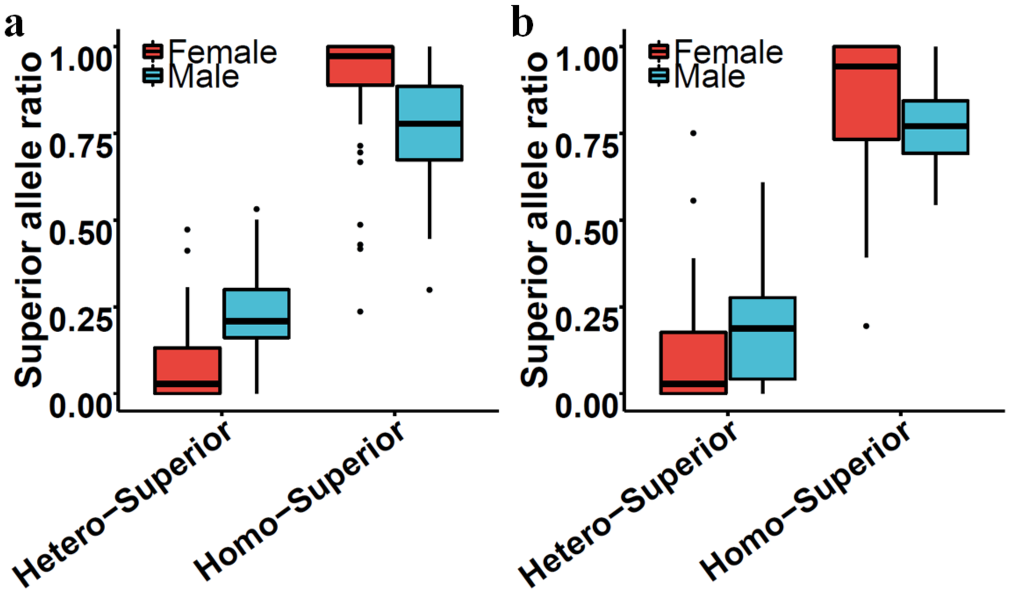

Supplementary Figure S27: Superior allele ratios of male sterile lines, restorer lines, and F1 hybrids in positive-non-additive (a) and negative-non-additive (b) QTLs identified in the SCA GWAS results.

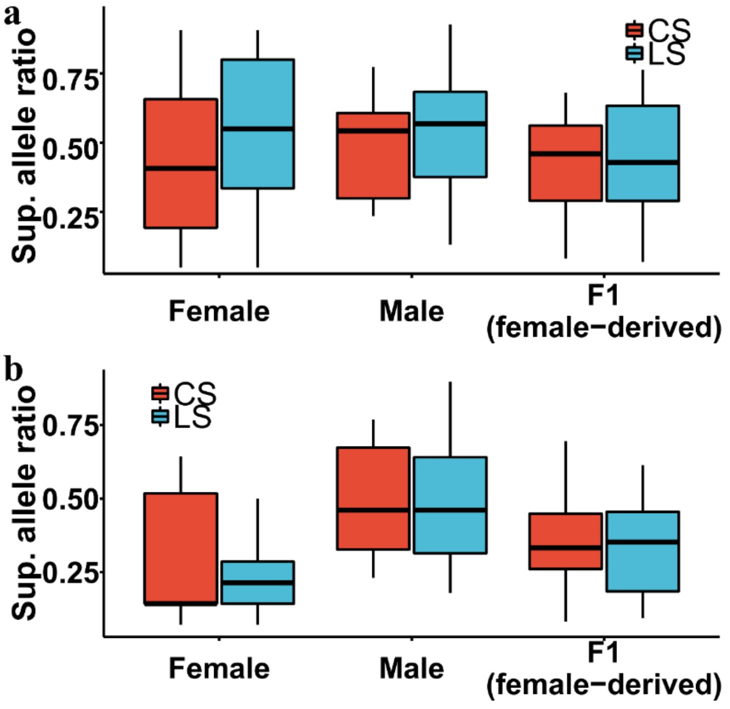

Supplementary Figure S28: Average MAF distributions around lead SNPs for three-genotype QTLs (a for CS, b for LS) and two-genotype QTLs (c for CS, d for LS) in additional 36 male sterile lines (Mat) and 48 restorer lines (Pat).

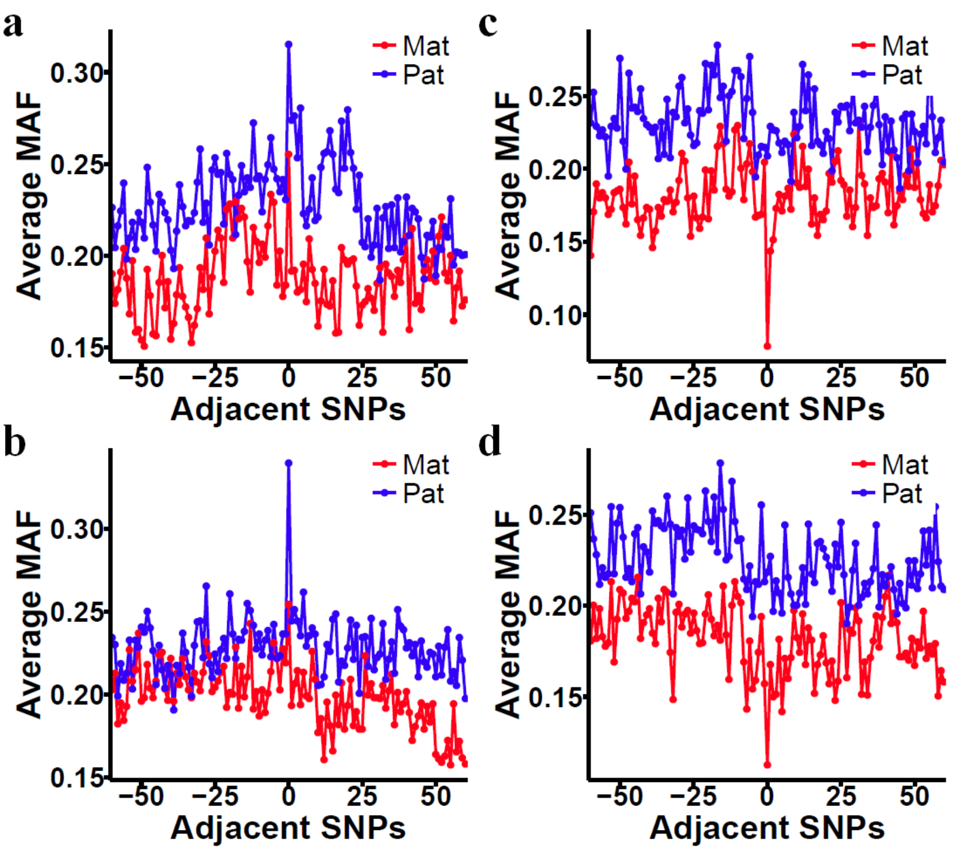

Supplementary Figure S29: Three-genotype QTLs were located in regions with increased nucleotide diversity in restorer lines. (a,b) Average Tajima's D distributions around lead SNPs of three-genotype QTLs identified in CS (a) and LS (b). (c,d) Average Tajima's D distributions around lead SNPs of two-genotype QTLs identified in CS (c) and LS (d).

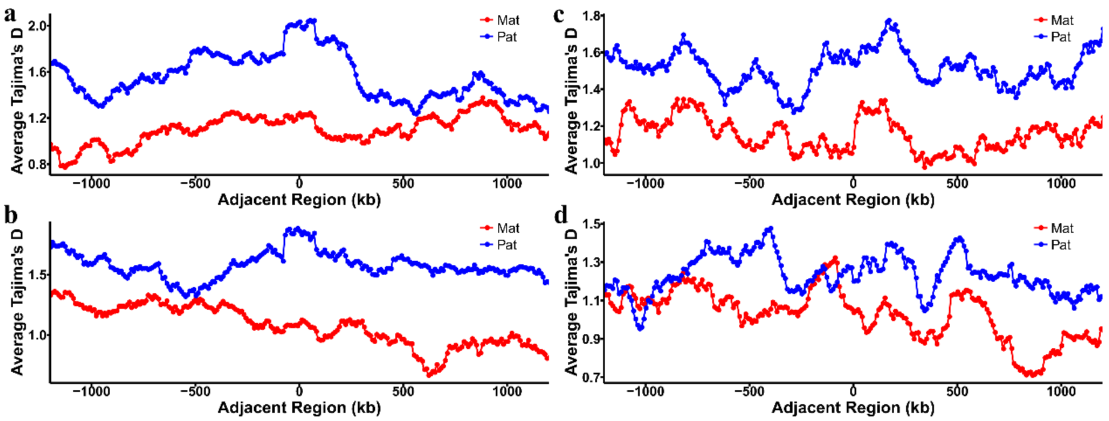

Supplement: Supplementary file 1 — Supplemental File [file 41598_2017_6742_MOESM1_ESM.pdf]
